# Mutational signatures are markers of drug sensitivity of cancer cells

**DOI:** 10.1038/s41467-022-30582-3

**Published:** 2022-05-25

**Authors:** Jurica Levatić, Marina Salvadores, Francisco Fuster-Tormo, Fran Supek

**Affiliations:** 1 Genome Data Science, Institute for Research in Biomedicine (IRB Barcelona), The Barcelona Institute of Science and Technology, C/ Baldiri Reixac 10, 08028 Barcelona, Spain; 2 Catalan Institution for Research and Advanced Studies (ICREA), Passeig de Lluís Companys 23, 08010 Barcelona, Spain; 3 Present Address: MDS Group, Josep Carreras Leukaemia Research Institute, Ctra de Can Ruti, Camí de les Escoles s/n, 08916 Badalona, Spain

**Keywords:** Targeted therapies, Data mining, Drug screening, Pharmacogenomics, Cancer genomics

## Abstract

Genomic analyses have revealed mutational footprints associated with DNA maintenance gone awry, or with mutagen exposures. Because cancer therapeutics often target DNA synthesis or repair, we asked if mutational signatures make useful markers of drug sensitivity. We detect mutational signatures in cancer cell line exomes (where matched healthy tissues are not available) by adjusting for the confounding germline mutation spectra across ancestries. We identify robust associations between various mutational signatures and drug activity across cancer cell lines; these are as numerous as associations with established genetic markers such as driver gene alterations. Signatures of prior exposures to DNA damaging agents – including chemotherapy – tend to associate with drug resistance, while signatures of deficiencies in DNA repair tend to predict sensitivity towards particular therapeutics. Replication analyses across independent drug and CRISPR genetic screening data sets reveal hundreds of robust associations, which are provided as a resource for drug repurposing guided by mutational signature markers.

## Abstract (web summary)

Mutational signatures can reveal the impact of mutagenic processes in cancer, including exposure to therapy. Here, the authors develop an approach that can accurately predict drug responses in cancer using mutational signatures while simultaneously correcting for germline variants with an ancestry matching procedure.

## Introduction

Cancer precision medicine draws on the presence of somatically acquired changes in the tumor, which serve as predictive markers of response to drugs and other therapies. Commonly these markers are individual genetic changes, such as driver mutations affecting oncogenes or tumor suppressor genes, or copy-number alterations thereof. Many commonly employed cancer drugs act by interfering with DNA synthesis or maintenance or by damaging DNA. Therefore, the altered capacity of cancer cells to repair and/or replicate DNA is the basis of many classical therapies, such as platinum-based agents, and also recently introduced or upcoming therapies, such as PARP inhibitors or ATR inhibitors (reviewed in refs. ^1–3^). It is paramount to identify predictive markers that are associated with failures of DNA maintenance in cancer cells.

However, while DNA repair is often deficient in tumors, many DNA repair genes such as *MLH1*, *MGMT*, *BRCA1,* or *ATM* do not commonly bear somatic mutations. Instead, they are commonly inactivated epigenetically^4–6^, or by alterations in trans-acting factors^7^, and so their deficiencies are difficult to predict from the gene sequence. Additionally, germline cancer-predisposing variants commonly affect DNA repair genes^8–10^, however, pathogenicity of such variants is often challenging to predict. Because of the above, other types of molecular markers may be more useful to infer about failed DNA repair. This is exemplified in ”BRCAness” – a gene expression signature that suggests a deficient homologous recombination (HR) pathway, even in the absence of deleterious genetic variants in the *BRCA1/2* genes.

In addition to gene expression, mutational signatures–readouts of genome instability–can characterize DNA repair deficiencies. One common type of signature describes relative frequencies of somatic single-nucleotide variants (SNV) across different trinucleotide contexts. Certain mutational signatures were found to be associated with failures in DNA mismatch repair (MMR) and HR pathways^11^ as well as DNA polymerase proofreading^12,13^ and base excision repair (BER)^14–16^ and nucleotide excision repair (NER)^17^ failures. Inducing DNA repair deficiencies in cancer cell lines is able to reproduce some of these signatures^18–21^. Other types of mutation signatures based on small insertions and deletions (indels)^9^ and on structural variants^22^ are also starting to be introduced.

Because mutational signatures describe the state of the DNA repair machinery of a cancer cell, they may be able to serve as a drug sensitivity marker. This is exemplified by a mutational signature associated with pathogenic variants in *BRCA1* and *BRCA2* genes^11,23^, thus identifying HR deficient tumors. The signature is common in ovarian and breast cancers, but genomic analyses have detected it across other cancer types^24,25^, suggesting the potential for broad use of drugs that target HR-deficient cells, such as PARP inhibitors. To this end, genomics-based predictors that draw on mutational signatures of HR deficiency have been developed^26,27^. We propose that this principle may extend to other types of mutational processes, potentially revealing tumor vulnerabilities.

Human cancer cell line panels provide an experimental model for the diversity in tumor biology that is amenable to scaling-up. Drug screens and genetic screens on large cell line panels^28,29^ have identified correlations between the sensitivity to a drug (or to a genetic perturbation), and the genetic, epigenetic, or transcriptomic markers in the cell lines. Encouragingly, genetic markers known to have clinical utility (e.g. *BRAF* mutations for vemurafenib, *EGFR* mutations for gefitinib, *BCR-ABL* fusion for imatinib sensitivity) are also evident in cell line panel data analyses^30^, suggesting potential for discovery of further useful genomic markers.

**Fig. 1:**
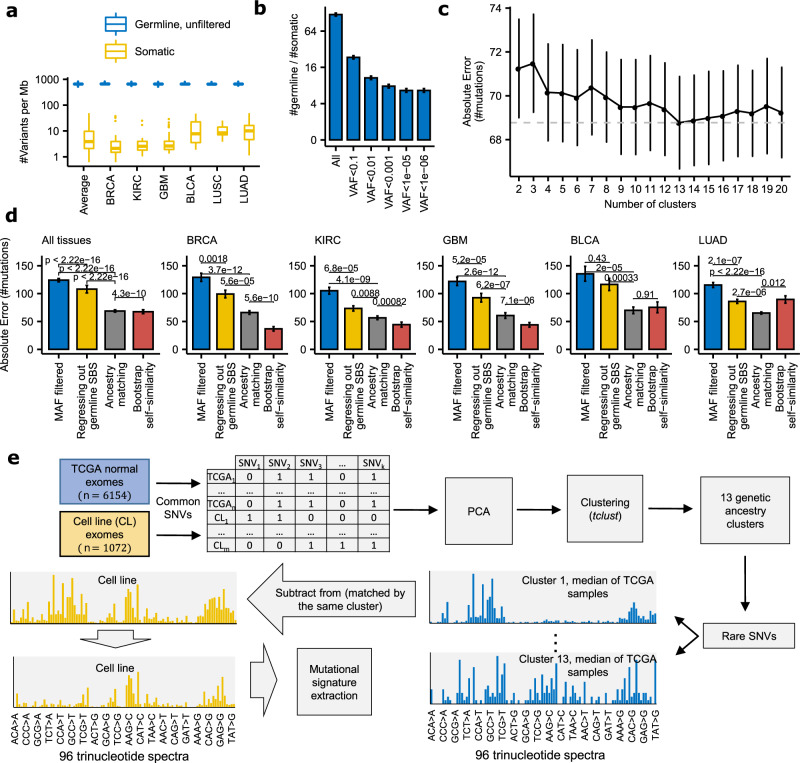
Evaluation of the ancestry-matching method to infer somatic mutation spectra on exomes without a matched normal control. **a**, **b** Germline variants greatly outnumber somatic mutations in exomes of various tumor types (*n* = 52 BRCA, 33 KIRC, 53 GBM, 19 BLCA, 15 LUSC, and 67 LUAD cancer exomes) (**a**), also after attempting to filter out germline variants according to the minor allele frequency (MAF) of variants listed in the gnomAD database (*n* = 450 cancer exomes) (**b**). The center line of box plots denotes medians of data points and the box hinges correspond to the 1st and 3rd quartiles, while whiskers extend to 1.5× IQR from the hinges. Data points beyond the end of the whiskers are shown individually. **c** Error between the real somatic 96 tri-nucleotide profiles and the profiles obtained with the ancestry-matching procedure, after various numbers of clusters (based on principal components of common germline variants; see Methods) are considered (*n* = 450 cancer exomes). **d** Comparison of the ancestry-matching method (with the number of clusters set to 13), the baseline procedure (variant filtering by population MAF<0.001%), regressing out mutational signatures reported as related to germline variants (signatures 1 and 5, and SNP signature^31,110^), and the error expected by chance (estimated by bootstrapping mutations). *P*-values by two-sided Wilcoxon rank-sum test (*n* = 450 cancer exomes). **e** A schematic representation of the ‘ancestry matching’ procedure. For compactness, the X-axes on the mutation spectra illustrations list only a subset of mutation types. PCA, principal components analysis. Error bars in panels **b**–**d** are the standard error of the mean. Source data are provided as a Source Data file.

Here, we used large-scale cell line data to investigate the hypothesis that mutational signatures in cancer genomes constitute markers of drug sensitivity. Quantifying somatic mutational signatures in cell line genomes is however difficult, because a matched normal tissue from the same individual is typically not available and thus cannot be used to remove the abundant germline variation. After filtering the known germline variants listed in population genomic databases^31,32^, somatic mutations are still greatly outnumbered by the residual germline variants (Fig. 1a, b), which may confound downstream analyses such as the inference of mutational signatures. We introduce a method to infer somatic mutational spectra from cancer genomes without a matched control sample, while adjusting for the residual germline variation. We apply this to infer trinucleotide mutation signatures in cancer cell line exomes, and identify associations with sensitivity to drugs and to genetic perturbation across cell line panels. Replication analyses across independent data sets indicated that mutational signatures are broadly applicable markers of drug sensitivity, matching or exceeding common genomic markers such as oncogenic driver mutations or copy number alterations.

## Results

### An ancestry-matching approach removes subpopulation-specific trinucleotide spectra to accurately infer mutation signatures

A substantial amount of the germline variation in a cell line exome cannot be removed by filtering based on minor variant frequency in population databases (Fig. 1b). Therefore we devised an approach to measure the somatic trinucleotide mutation spectrum – the input for the inference of mutational signatures^33^ – while rigorously adjusting for the contamination by the residual germline mutation spectrum.

Because mutational processes differ across human populations^34^, there is potential for this to confound analyses of the somatic mutation spectrum. Given the high number of residual germline variants post-filtering (Fig. 1b), even slight differences in the germline spectrum can cause large deviations in the observed spectrum, which is a mix of somatic and germline variation.

To address this, we implemented an ancestry-matching procedure, looking up the individuals with a similar ancestry to each cell line’s ancestry. In particular, we clustered the cell line exomes together with germline exome samples from the TCGA data set, grouping by principal components derived from common germline variation (Fig. 1e; Methods section). The TCGA individuals clustered with a cell line provided a baseline germline mutational spectrum, which can be subtracted from the observed mutation spectrum to estimate the somatic mutation spectrum.

We benchmarked our ancestry-matching procedure for the accuracy of reconstructing the correct somatic mutation spectrum in a cancer cell line exome. To this end, we used SNV calls from TCGA cancer exomes where the matched normal was ignored, thus simulating the mutation calls that would be obtained from cell line genomes (see Methods section). We then compared the reconstructed somatic SNV mutation spectrum to the true somatic spectrum, obtained by contrasting tumor exomes with the matched healthy tissue exomes from the same individuals.

Ancestry-matching improves over the commonly used strategy, that is simply filtering out known germline variants according to population genomic databases (Fig. 1c, 1d and Supplementary Fig. 1d); error in somatic trinucleotide frequency spectrum (Methods section) is 68.8 versus 124.1 across all tissues, while for comparison, the error expected by ‘self-similarity’ via a bootstrap-resampling of mutations from the same tumor samples would be 67.5, close to that obtained via ancestry-matching.

We considered various numbers of population clusters according to the error in reconstruction of the correct somatic trinucleotide spectrum. Selecting three clusters, expectedly, recovers the major ethnicity groups (European, Asian and African, Supplementary Fig. 1a) and further increasing the number of clusters to 13 minimizes the error in reconstructing true somatic trinucleotide mutation spectra (Fig. 1c; error is 68.8 for 13, versus 71.5 for 3 clusters; this improvement is modest and so the 3-cluster solution may also provide a satisfactory baseline for downstream mutational spectra analyses).

Encouragingly, comparing the 13 ancestry clusters sorted by self-reported ethnicity (Supplementary Fig. 1b), intra-ethnicity trinucleotide mutational profiles are more similar than the inter-ethnicity profiles (Supplementary Fig. 1c), and a PC analysis of the trinucleotide spectra of the rare variants separates the major ethnicity groups (Supplementary Fig. 1d). This is consistent with reports of differential mutagenic processes in the human germline across ancestral groups – for example, the HCC>HTC (H = not G) variants were reported to be increased in Europeans, NCG>NTG mutations in Native Americans and NAC>NCN and TAT>TTT in some East Asians^34–36^. These reports, together with our benchmarking using simulations, support the use of ancestry-specific baselines in inferring somatic mutational spectra of unmatched cancer genomes, such as cell line genomes.

We applied the ancestry-matching methodology (Fig. 1e) to exome sequencing data of 1071 cancer cell lines^37^, yielding their somatic trinucleotide spectra. On this data, we performed de novo discovery using an NMF approach, broadly as described by Alexandrov et al.^33^ (with certain modifications, see Methods section), where we extracted those NMF solutions that resembled previously reported tumor mutational signatures^9^ of single base substitutions (SBS). We tested a number of variations on the data filtering and the mutation extraction methodology (Supplementary Data S1) to improve agreement with the known set of SBS signatures^9^ and their known distribution across tissues, as well to improve power of the set of mutational signatures to predict drug responses in the cell lines (Methods section; Supplementary Data S1). To further demonstrate the utility of the ancestry matching approach in combination with NMF signature extraction, we again used the set of simulated cell line exomes as above, where the true somatic mutation signatures are known because the matched-normal was available. The ancestry-matching significantly improves the cosine similarities towards true NMF signature spectra, compared with the usual approach of filtering population variants (*p* = 0.021, Wilcoxon test; Supplementary Fig. 1g) and similarly so for the signature exposures (*p* = 0.017; Supplementary Fig. 1g). We conclude that our implementation of ancestry-matching benefits NMF mutation signature extraction in unmatched cancer samples; we recognize that future variations on this methodology might bring improvements.

We jointly inferred trinucleotide (or SBS) signatures together with a set of indel mutational features. Examining the SBS part of the spectrum, this yielded 30 cell line mutational signatures that very closely match (at a cosine similarity cutoff ≥0.95) the known tumor SBS signatures, and a further 22 cell line signatures that match known SBS tumor signatures (at a stringent cosine similarity ≥0.85 and <0.95; a randomization test estimated that a ≥0.85 cosine threshold corresponds to a 1.8% FDR in matching the correct SBS, Supplementary Fig. 4b).

The former group was labeled with the name of the corresponding SBS signature, while the latter similarly so plus the suffix “L” (for “like”). In some cases, our cell line signatures were similar to more than one previous tumor SBS (Supplementary Fig. 4a) and they were named such as to make this evident, for instance our SBS26/12L matches the DNA mismatch repair (MMR) failure signature SBS26 and a possible MMR failure signature SBS12^38^ (more similar signature listed first). Note that a comparable degree of ambiguity is also observed among some of the known tumor SBS mutational signatures (Supplementary Fig. 5). The full set of 52 mutational signatures we inferred and their ‘exposures’ across cell types are visualized in Supplementary Figs. 2 and 3, and corresponding data is provided as Supplementary Data S2 and S3.

Additionally, there were five mutational signatures that appeared specific to cell lines (SBS-CL), meaning they did not closely match one of the signatures from current tumor catalogs (Supplementary Fig. 2 and Supplementary Data S2). These mutational processes may be evident only in rare tumor types or they may be active predominantly in cultured cells rather than in tumors. Some might originate from the incomplete separation of other signatures (Supplementary Fig. 6 shows examples). Finally, some SBS-CL may reflect contamination with residual germline variation, as well as with sequencing artifacts, similarly as was recently reported for many SBS signatures recovered from tumor genomes^9^.

### Mutational signatures predict cell line drug response more accurately than oncogenic mutations or copy number alterations

Genetic and epigenetic alterations in cancer cell lines are often investigated as markers of sensitivity to chemical compounds^29,30^. We hypothesized that mutational signatures in a cell line genome can serve as similarly informative markers of drug sensitivity or resistance. We compared their predictive ability to that of the markers commonly used to predict drug response in cell lines: oncogenic mutations (in 470 cancer driver genes^30^), recurrent focal copy number alterations (CNAs at 425 genes^30^), and DNA methylation data at informative CpG islands (HypMet at 378 genes^30^). Additionally, we examined gene expression patterns (mRNA levels of 1564 genes that are either represented in the L1000 assay^39^ or are known drug target genes^40^), because gene expression can be highly predictive of drug response^30,41^, possibly because it reflects differences between various cancer types and subtypes.

**Fig. 2:**
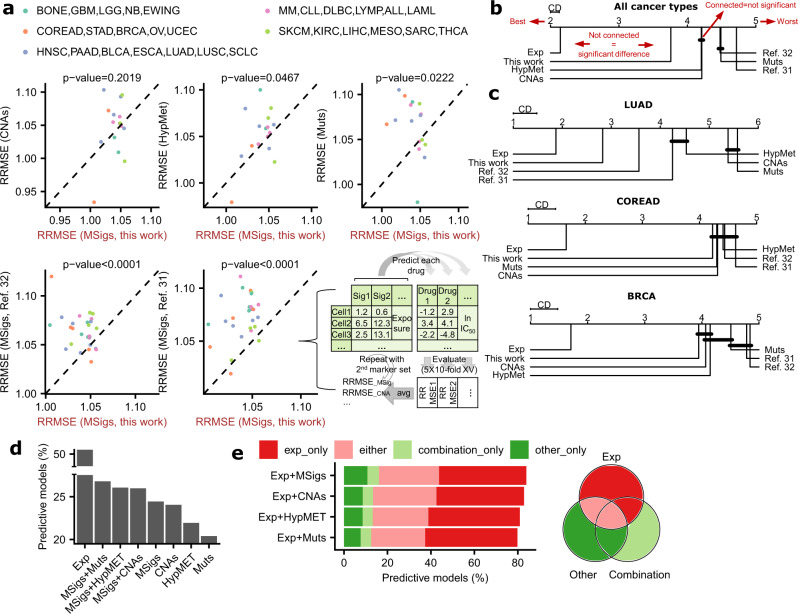
Prediction of drug response with mutational signatures and other molecular data types. **a** Predictive performance (RRMSE, relative root-mean-square error) of drug response prediction with mutational signatures (“MSigs") reported here and previously^31,32^ and other data types (oncogenic mutations (“Mut”), copy number alterations (“CNAs”) and DNA hypermethylation (“HypMet”)). P-values of paired one-sided Wilcoxon signed rank test are reported on the plots. Dashed line denotes the diagonal. Bottom right panel shows a schematic of how RRMSE for each tissue was estimated, where “Sig” is mutational signature or other marker (CNA etc.), “Cell” is cell line, “XV” is crossvalidation, and “Predict” implies a Random Forest model. **b** Average rank diagrams for the performance (RRMSE) of the drug response prediction from various sets of markers, using Random Forest. “This work” refers to the mutational signatures inferred here, while “ref. ^31^.” and “ref. ^32^.” refers to prior sets of mutational signatures. Each graph shows: the ranking among the different marker sets (those at the left-hand side are the best performing) and the significant differences between pairs of marker sets (if their ranks are at least critical distance (CD) apart, the difference in predictive performance is statistically significant at *p* < 0.05, by the Nemenyi post-hoc test, two-sided). The groups of marker sets for which there is no significant difference are connected by black lines. **c** Tests shown separately for four tissues-of-origin with the highest number of cell lines in our panels. **d** The percentage of the Random Forest models that are predictive of drug response, defined as having a predictive error lower than the one of an uninformative default model (predicts the average log IC50 for every cell line). Expressed relative to the total number of testable drug-tissue pairs. **e** The percentage of models that are predictive with gene expression but not another feature type (“exp_only”), by other feature type but not with gene expression (“other_only”), by either model (“either”), or only by a combination of both (“combination”). Source data are provided as a Source Data file.

We predicted the sensitivity (log IC50 concentration) of a panel of 930 cell lines (separately for 29 cancer types that had a sufficient number of cell lines available) to a set of 518 drugs from the GDSC database^37^. In particular, we used Random Forest (RF) regression applied to the complete set of genetic or epigenetic markers (listed above) in an individual cell line as features (Fig. 2a). In addition to mutational signatures inferred herein, we also considered the cell line mutational signatures reported by two recent studies^31,32^, obtained using approaches that did not account for ancestry and that have moreover fit the data to pre-existing sets of SBS signatures, rather than extracting signatures de novo from cell line genomes (see Methods section).

Firstly, mutational signatures predicted drug sensitivity significantly better than all other tested types of alterations: the increase in accuracy of RF models over CNAs, DNA hypermethylation, and oncogenic mutation features is significant (at *p* < 0.05, by corrected Friedman test followed by the post-hoc Nemenyi test on ranks; Fig. 2b; Methods section; average rank for mutational signatures was 3.75 while for other types of (epi)genetic features it was 4.20–4.49, considering all RF models). Secondly, mutational signatures found by our ‘ancestry matching’ approach perform significantly better than other cell line signatures recently reported^31,32^, comparing across the set of cell lines that overlap between the publications (Fig. 2b, c). Moreover, these previous sets of cell line mutational signature exposures were less predictive of drug sensitivity than were CNAs, oncogenic mutations and DNA methylation, suggesting the utility of adjustment for germline spectra contamination prior to mutational signature inference (Fig. 2b–d).

Next, we applied a different test that considers the average error in predicting the drug sensitivity profile (relative RMSE of a RF model in crossvalidation; Fig. 2a), averaged across all drugs in a given tissue. In most cancer types, the mutational signatures obtained herein were better predictors of drug response, than all the usual genomic and epigenomic features (13 out of 16 tested cancer types compared with DNA hypermethylation, 10 out of 16 for CNAs, and 10 out of 16 for oncogenic mutations). Also, in most cancer types (Fig. 2a) our cell line signatures significantly outperformed recent methods to infer mutational signatures^31,32,42^ naive to germline mutational spectra (in 22 and 25 out of 27 cancer types for the two previous methods that used the same set of cell lines, Fig. 2a, and in 11 out of 16 cancer types on a set of overlapping cell lines for a third method (Supplementary Fig. 7a); *p* < 0.0001, *p* < 0.0001, and *p* = 0.041, respectively, Wilcoxon test for decrease of relative RMSE).

Gene expression was overall very highly predictive of drug response, (Fig. 2b–d), consistent with recent reports^30,41^. We asked if the predictive power of gene expression can be complemented by additionally including mutational signatures and/or various sets of genetic markers. We predicted the drug response profile in RF models as above (Fig. 2a), but here by using combinations of marker types with gene expression, and tallying the predictive RF models (drug-tissue pairs with better-than-baseline RRMSE in crossvalidation; Methods section). Notably, gene expression is complemented by mutational signatures and also by other types of features, yielding a higher percentage of predictive RF models when markers are combined than with gene expression alone (Fig. 2d, e). If gene expression markers are unavailable, the mutational signatures were still complementary to oncogenic mutations, CNAs, or DNA methylation (Fig. 2d).

Next, we considered the complementarity analyses at the level of individual drugs, asking if the profile of drug sensitivity that can be predicted by a combined RF model (e.g. gene expression and mutational signatures) could also have been predicted by the two RF models drawing on the individual sets of features – on gene expression only, or on mutational signatures only (Fig. 2e). The number of RF models where gene expression by itself is not predictive but mutational signatures are predictive is substantial (891 drug-tissue pairs, plus 432 where only a combination of signatures and expression is predictive). This is higher than the number of drug-tissue pairs where gene expression is not predictive but driver mutations are (563 plus 352), and similarly so for CNA (633 plus 347). In addition to gene expression, using DNA methylation as a baseline also supports that response profiles for many drug-tissue combinations can be predicted only by mutational signatures (Supplementary Fig. 7b). This suggests that the predictive signal in mutational signatures does not simply reflect cancer subtype or cell-of-origin, at least to the extent that subtype can be identified via gene expression or DNA methylation patterns. Overall, mutational signatures, considered collectively, can complement gene expression and other types of markers in predicting drug sensitivity profiles of cancer cells.

### Associations with drug response that replicate in independent data sets

**Fig. 3:**
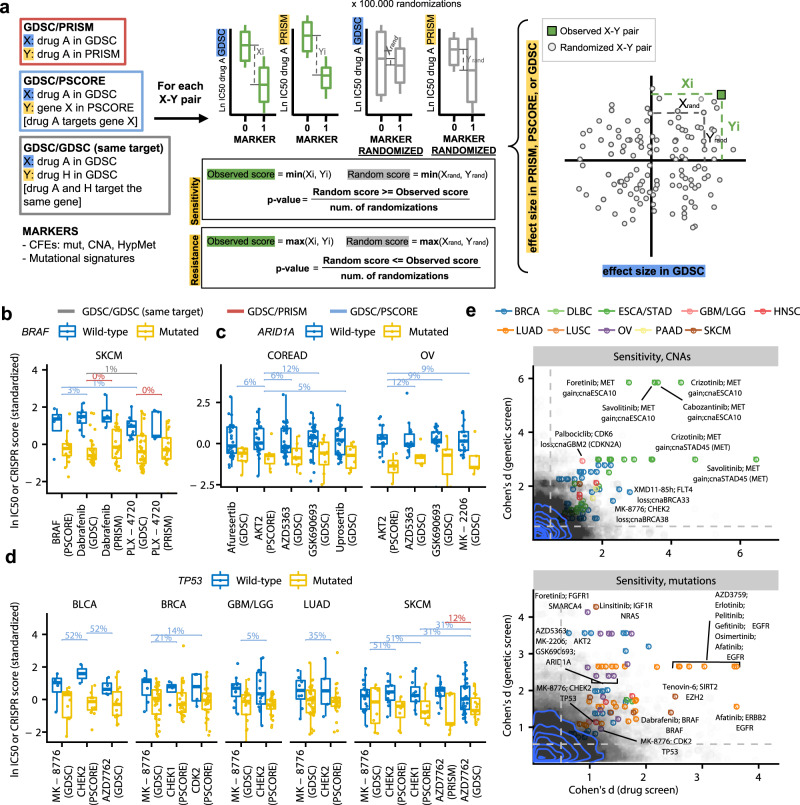
Detecting robust associations between genetic or epigenetic markers and drug sensitivity by replication across measurements. **a** A schematic of the randomization test methodology to detect replicating associations using three different tests: (i) consistent effects of a drug between two screening assays (GDSC/PRISM), (ii) effects of a drug consistent with effects of knockout of the target gene (GDSC/PSCORE), and (iii) effects consistent across different drugs that share the same molecular target (GDSC/GDSC or “same target”). Box plots and scatterplot are illustrative. **b**–**d** Examples of replicated associations of a known example of oncogene addiction (to *BRAF*, b) and of additional cancer vulnerabilities associated with mutations in tumor suppressor genes (*ARID1A*, **c**; *TP53* **d**). Y-axes show a Z-score derived from either the ln IC50 value (i.e. drug sensitivity, in columns labeled “GDSC” or “PRISM”) or from the CRISPR essentiality score (in the column labeled “PSCORE”). Horizontal brackets show FDR for replicated significant difference between *wild-type* and mutant genotypes, obtained via a randomization test in panel **a**, where color denotes the type of the replication test (“GDSC”, “PRISM” or “PSCORE”). The center line of box plots denotes medians and the hinges correspond to the 1st and 3rd quartiles, while whiskers extend to 1.5× IQR from the hinges. e, Effect sizes of markers that associate with drug response in the GDSC drug screen (X-axes) and with response drug target gene knock-out in the Project SCORE genetic screen (Y-axes), shown separately for copy number alterations (CNA) and mutations in cancer genes (Muts). Gray points represent all tested associations, while colored points denote the statistically significant associations that also meet an effect size threshold. Blue lines are the contours of the 2D kernel density estimates. Representative points are labeled. Drug names on grouped labels (“Muts” sub-panel) are ordered by their appearance on the plot from left to right. Source data are provided as a Source Data file.

Because of reproducibility concerns in large-scale drug screens^43–45^ that might stem from technical reasons or from cancer cell line evolution during culture, we asked if the associations between mutational signatures and drug responses replicate across data sets. To this end, we tested for associations involving various markers, with the additional condition that the associations also replicate in an independent data set. We implemented a randomization-based procedure that tests that the smallest effect size (Cohen’s *d* statistic) across both datasets is above chance (Fig. 3a; Methods section). A value of *d* ≥ 1, typically considered a large effect size, implies that a difference in mean drug sensitivity (log IC50) between the cell lines positive for a marker and those negative for a marker is greater than the pooled standard deviation (of the log IC50) of the two sets of cell lines. These replication tests were performed on binarized mutational signatures i.e. the signature present/absent indicator variables (Supplementary Fig. 15b), considering different cancer types individually (Supplementary Data S4). Additionally the same tests were performed on the usual markers in cell line screening analyses, including oncogenic driver mutations (Fig. 3b–e), CNAs (Fig. 3e), and promoter DNA methylation^30^.

We considered three different types of replication analyses: an external replication in an independent drug screening data set, internal replication with multiple drugs affecting the same target, and an external replication using CRISPR/Cas9 gene knockout fitness screening data. Randomization p-values from these three replication methods (across various tissues) were rarely inflated for mutational signatures, and in fact commonly exhibited deflation (mean lambda across tissues 0.68–1 for different replication methods, Supplementary Fig. 8) suggesting an overall conservative bias in the replication test as implemented. The few tissue-method combinations that did exhibit inflation in *p*-values (lambda >1.3; Supplementary Fig. 8) were omitted from further analyses of the associations; the full set of associations are nonetheless included in the Supplementary data, for completeness.

**Fig. 4:**
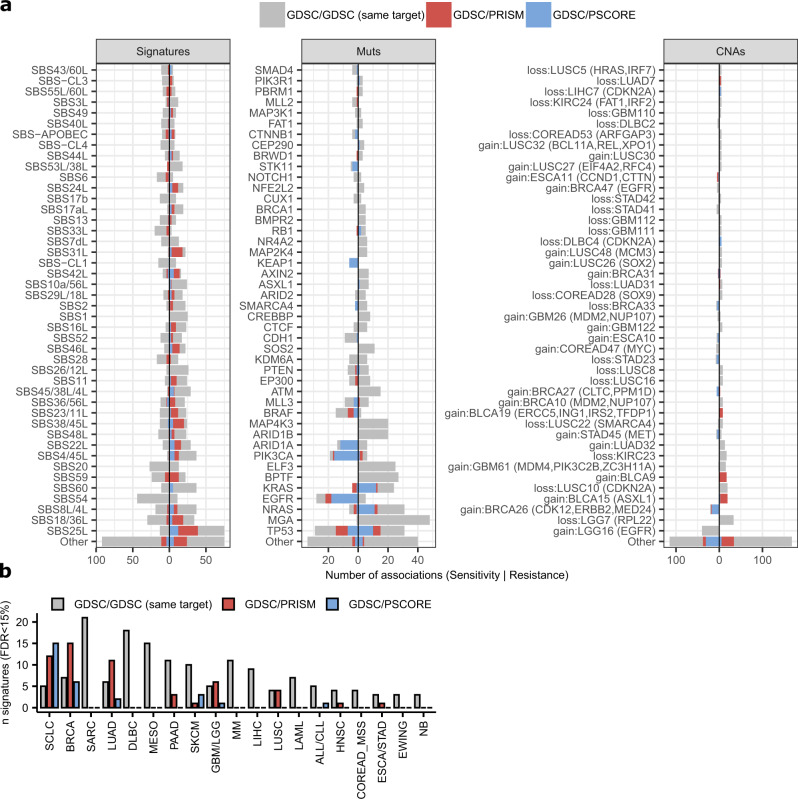
Tally of the significantly replicated associations of drug sensitivity or resistance with mutation signatures and other markers. **a** Comparison of the number of statistically significant associations (FDR<15% by randomization test, additionally requiring an effect size *d* > 0.5 for GDSC/PRISM and GDSC/PSCORE tests, and *d* > 1 for the GDSC/GDSC (same-target) test) per feature, among mutational signatures (“Signatures”), oncogenic mutations (“Muts”) and copy number alterations (“CNAs”) in the three types of replication tests (see Fig. 3a). Features are ranked by the total number of significant associations, either for drug sensitivity (negative side of X-axis) or resistance (positive side of X-axis). **b** The number of different mutational signatures that have statistically significant associations across various cancer types (at FDR<15%; we consider signatures that have >1 significant association per cancer type), in the three replication tests. Source data are provided as a Source Data file.

Firstly, we performed a replication analysis where the drug association data from the GDSC was tested against another drug screening data set: PRISM (derived from an experimental methodology based on pooled barcoded screens^46^; 348 cell lines and 178 drugs overlap with the GDSC set). In total, 290 drug-mutation signature associations were robustly supported across both GDSC and PRISM (*d* ≥ 0.5 and same direction of effect in both datasets and additionally requiring randomization test FDR<15%; adjustment using the *q*-value method^47^), observed across diverse tissues and diverse signatures (Fig. 4a, b and Supplementary Fig. 12a, b). This exceeds the number of drug associations replicated in PRISM involving driver mutations (37), copy-number changes (55), or DNA methylation (64) in the same test. We list the associations in Supplementary Data S5.

Given that the amount of cell lines available to the replication analysis is reduced and thus statistical power is limiting, particularly for some tissues (Supplementary Fig. 13b), we suggest that some associations at permissive thresholds (here, nominal *p* < 0.005) might be of interest for use as supporting evidence, corroborating other associations (see below).

Secondly, we performed an internal replication analysis within GDSC, enforcing that associations must be detected with two or more drugs that share the same molecular target. In total, 228 drugs in the GDSC data could be tested in this “same-target” analysis. Effectively, multiple drugs serve as pseudoreplicates, and additionally this test may help discard associations due to off-target effects, which are more likely to differ between two drugs than their on-target effects. Here, we identified 971 significant associations for mutational signatures, 206 for driver mutations, 288 for copy-number changes, and 762 for promoter DNA methylation (at effect size *d* > 1 and FDR < 15%) (Fig. 4a, b; data in Supplementary Data S7 and S8 for the associations between the default FDR<15% threshold, and the permissive threshold at *p* < 0.005, respectively).

Some associations overlapped between the same-target and the GDSC-PRISM replication analyses, suggesting more robustness: Supplementary Data S8 contains those replicated associations that were seen either across multiple cancer types, and/or across multiple drugs that target the same pathway, and/or across different replication methods (including also CRISPR genetic screens, see below). We suggest that this ‘silver set’ of 3911 associations, where each replication at a FDR < 25% is further supported by one or more additional replications at a suggestive (nominal *p* < 0.005) threshold, to be potentially suitable for further analyses or follow-up.

### Integrating drug screening data with genetic screening data to obtain robust associations

As a third type of replication analysis, we prioritized cancer vulnerabilities by intersecting the drug sensitivity data with genetic screening data sets. Our rationale was that a biological process may be targeted similarly by pharmacological inhibition of a protein, or by editing the genes that encode the corresponding protein. In specific, we examined sensitivity to CRISPR/Cas9-mediated knockouts that target the protein-coding genes, across a panel of 517 cell lines^48^ that overlapped the GDSC cell lines. We identified many associations (at effect size *d* > 0.5 and FDR<15%) with conventional markers – oncogenic driver mutations (*n* = 123), copy number alterations (*n* = 100), and DNA methylation (*n* = 86) – that replicated across drug and genetic data (Supplementary Data S6, Figs. 4 and 3e, and Supplementary Fig. 9b, c).

Demonstrating the utility of this test, we recovered well-known examples of the oncogene addiction paradigm, such as the breast and esophageal/gastric cell lines with *ERBB2* (*HER2*) amplification being sensitive to inhibitors of EGFR and ERBB2 (afatinib and 4 other drugs), and also to the *ERBB2* gene knockout (replication FDRs all <15%). Similarly, we recapitulated the known associations between amplifications of a chromosomal segment 7q31 including the *MET* oncogene, and sensitivity of esophageal/gastric cancer to crizotinib^49,50^ and also 3 other MET inhibitors (FDRs < 6%; Fig. 3E). The *BRAF* mutations in skin and colorectal cancer likewise sensitize to different BRAF inhibitors in both the GDSC and PRISM drug data, and also to *BRAF* gene disruption (Fig. 3b and Supplementary Fig. 9d). Conversely, we note that some oncogene mutations can also confer drug resistance e.g. *NRAS*-mutant leukemia cells (Supplementary Fig. 10, *n* = 38 hits in Fig. 4a), consistent with prior reports (discussed in Supplementary Note 1). These and other striking associations with gene mutations, CNA, and promoter DNA methylation that replicated in the genetic screening data are highlighted in the global data overview in Fig. 3e and Supplementary Fig. 9c.

In addition to oncogene addiction, replicated associations can suggest ways to target mutated tumor suppressor genes via synthetic lethality. One example is a *CDKN2A* deletion that sensitizes brain cancer cells to palbociclib (a CDK4/6 inhibitor) and to knockouts in *CDK4* and *CDK6* genes. In contrast, *RB1* mutations were associated with resistance, consistent with the biological roles of these genes, as well as prior preclinical studies (details in Supplementary Note 1). This demonstrates the power of the joint analyses of drug and genetic screening data here and elsewhere^51^, suggesting that the other associations we identified here (Supplementary Data S6) provide cancer dependencies promising for follow-up. For example, our analysis identifies vulnerabilities of *TP53*-mutant cells to manipulating the activity of the CHK2/CDC25A/CDK2 axis across five different cancer types (Fig. 3d and Supplementary Fig. 9e), echoing prior work on therapeutic interventions on CHKs in *TP53*-deficient cells (Supplementary Note 1). These examples also illustrate how an integrated analysis of drug screening data with genetic screening data can reveal drug effects exerted via secondary drug targets (e.g. likely CHK2 for the MK-8776 inhibitor of CHK1; Fig. 3d, see discussion in Supplementary Note 1).

We highlight a robustly supported synthetic lethality example involving mutations in the *ARID1A* tumor suppressor gene and the inhibition of the *AKT2* gene or protein (Fig. 3c). In particular, *ARID1A* mutant colorectal cell lines are more sensitive to the knock-out of the *AKT2* gene by CRISPR, as well as to the pan-AKT inhibitors GSK690693 and capivasertib/AZD5363 (FDR = 6% and 12% in the replication test, respectively). The same is observed in ovarian cancer cell lines, again involving *AKT2* knockout and the same two inhibitors (at FDR = 9% and 12%, respectively). This is supported by additional AKT inhibitor drugs: afuresertib (FDR=6%), AKT inhibitor VIII (FDR = 21%), and uprosertib (FDR = 5%) in colon, and MK-2206 (FDR = 9%) in ovary (Supplementary Data S6). Further evidence for an interaction between these genes is found in tumor genomic analysis. The *AKT2* oncogene can be amplified in ovarian, endometrial, pancreatic and other cancer types, while the *ARID1* tumor suppressor commonly bears truncating mutations in many cancers. In tumor genomes, *AKT2* alterations significantly co-occur with *ARID1A* alterations (OR = 2.0, FDR<0.1% in MSK-IMPACT cohort of 10,945 samples;^52^ replicated at OR = 1.4, FDR<0.1% in an independent TCGA pan-cancer cohort of 10,967 samples; analysis via cBioPortal^53^). These genomic associations support that the *AKT2* amplifications may bring a selective benefit to *ARID1A*-mutant tumors. Overall, our analyses solidify the notion that the PI3K/AKT/MTOR signaling inhibition is a vulnerability of *ARID1A*-mutant cells^54–57^, as reported before for individual examples of cell lines sensitized to AKTi drugs upon silencing of *ARID1A*^56^, and we further suggest specifically AKT2 as an opportune point of intervention.

Next, we applied this same statistical methodology (Fig. 3a; Methods section) to identify replicated drug sensitivity associations involving mutational signatures in cell line genomes.

### Mutational signatures associated with sensitivity both to pharmacological and genetic perturbations

As a positive control in a study of mutational signatures as markers, we considered a recently reported vulnerability of cell lines that are microsatellite-instable (MSI) and therefore deficient in the DNA mismatch repair (MMR) pathway, which do not tolerate the loss of the *WRN* gene^48,58–60^. MMR deficiencies in tumors are known to associate with MSI and with trinucleotide mutational signatures SBS6, 15, 21, 26, and 44^9^ (and additionally SBS14 and 20 which result from MMR failure concurrent with deficiencies in replicative DNA polymerases). In a joint analysis of MSI-prone cancer types (colorectal, ovary, stomach, uterus), we found links between the MMR SBS signatures that we inferred in the cell line exomes and the sensitivity to *WRN* knockout. However, levels of statistical support were variable across the MMR signatures (FDRs <0.01%,<0.01%, <0.01%, 11%, 18%, 21%, and n.s. [associated with resistance]) for SBS20, 15, 6, 26/12L, 14, 21, and 44L, respectively; Supplementary Fig. 11a). Additionally, we noted some additional signatures with a high weight on the indel components – and thus might be MMR-related: SBS33L, SBS54, and SBS-CL1 (Supplementary Fig. 2) – also predicted sensitivity to *WRN* loss (Supplementary Fig. 11), in case of SBS33L with a high effect size. Thus, some MMR-associated signatures are more robust markers for WRN inhibition (particularly the C > T-rich SBS15 and SBS6, as well as SBS20) than the other MMR failure-associated signatures (such as the T > C rich 26, 21, or 44). Conceivably, this might be because these signatures reflect different types of MMR failure that confer differential requirements for WRN activity. Overall, the ability to recover the known *WRN* dependencies of MMR-deficient cell lines estimated via trinucleotide mutational signatures supports the utility of our methodology to infer mutational signatures in cell line genomes.

Beyond *WRN* disruption, the MMR signatures as well as other mutation signatures can predict sensitivity to many perturbations, including those that the MSI status nor the other genetic markers would not predict (Supplementary Fig. 9a; we note that the converse is also true, at least for the few cancer types where MSI labels are available).

Next, we systematically examined all mutational signatures for the overlap between associations in the GDSC drug screen and Project SCORE genetic screen. This yielded 130 associations (at a randomization FDR < 15%, and additionally requiring an effect size of *d* > 0.5 in both the genetic and the drug screens) that replicated across data sets – a higher number than for oncogenic driver mutations, CNAs, and DNA methylation (123, 100, and 86, respectively, at the same FDR threshold). These associations (Fig. 4, Supplementary Fig. 12c; full list in Supplementary Data S6) involved k.o. in 64 different genes, indicating that mutational signatures associate with a variety of target genes and suggesting potential points of intervention for follow-up. The number of replicated associations involving mutational signatures highly ranked by this replication analysis (such as the chemotherapy-associated SBS25L, the haloalkane exposure-associated SBS42L, and the signature possibly related to NER deficiency^61^ SBS8L/4L; Fig. 4b, Supplementary Fig. 12a; *n* = 12, 8, and 7 replicated associations at FDR 15%, respectively) broadly matches the number of replicated associations involving common driver mutations such as *EGFR* or *TP53* or *KRAS* (*n* = 18, 17, and 13, respectively), or known copy number change events such as *ERBB2* gain (*n* = 17 replicated associations in Project SCORE) (Fig. 4a and Supplementary Data S6). We also show the tally of associations at a more permissive 25% FDR in Supplementary Fig. 12, further supporting how mutational signatures provide markers as commonly associated with drug response as the usual markers based on driver mutations and CNA.

We note that in this and further analyses we have, conservatively, stratified colorectal cancer cell lines into MSI and MSS (association counts without stratification are in Supplementary Fig. 13a and Supplementary Data S9), based on MSI being common in colorectal cell lines, and MSI status being strongly associated with mutational signatures^9,38,62^.

### Robust associations involving mutational signatures replicate across multiple cancer types

We next focused on those drug associations involving mutational signatures that recurrently replicated across more than one replication method (see Fig. 3a) and/or more than one cancer type (‘silver set’, Supplementary Data S10). We noted that some associations in this set recurred in three or more methods and/or tissues, thus we introduced a more stringent tier of hits, with a higher priority for follow-up. These ‘golden set’ hits recurred in at least three different tissues or in three different tests with effect size *d* > 0.5 and significant at *p* < 0.005, and at least once at FDR < 25%. This resulted in 995 higher-priority associations (tallying both mutational signature associations, and the driver mutation and CNA associations; Supplementary Data S8).

**Fig. 5:**
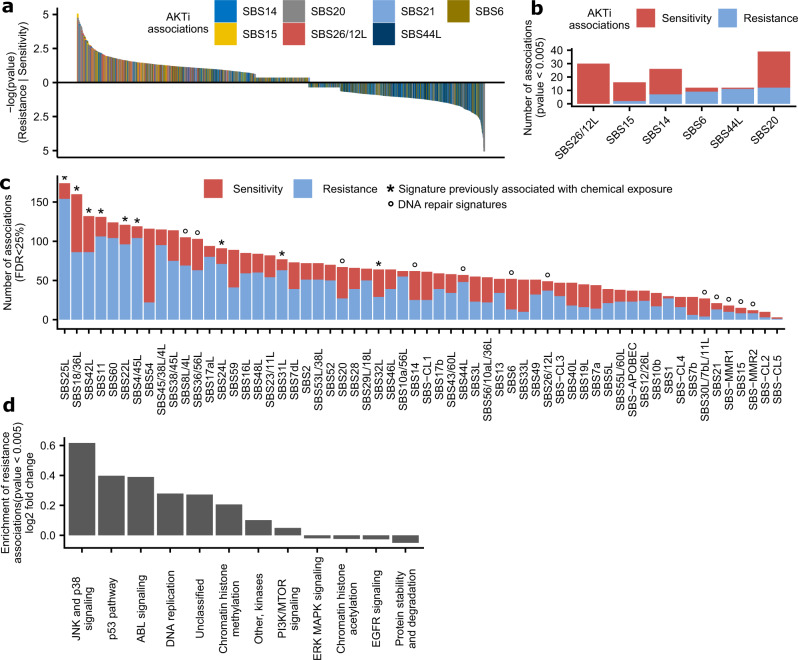
Highlighted examples of robustly supported associations involving mutational signatures. **a** All tested associations between AKT inhibitors and DNA mismatch repair mutational signatures, across all three replication tests (by the “two-way” randomization test, see Methods section). Each bar represents a *p*-value of one association. For associations with effect size <0.2, *p*-values were not calculated in the randomization procedure and are here shown as having *p* = 0.5. **b** Associations having *p* < 0.005 between AKT inhibitors and individual DNA mismatch repair signatures. **c** The tally of significant associations at FDR < 25% across all three replication tests. The * and ° symbols denote groups of mutational signatures; see key embedded within the panel. **d** The average log2 ratio of observed vs. expected frequencies of occurrence of drug target pathways in resistance associations (*p*-value < 0.005) with signatures of chemical exposure, over the top 6 signatures by the number of resistance associations (SBS25L, SBS18/36L, SBS42L, SBS11, SBS22L, and SBS4/45L). Source data are provided as a Source Data file.

A common occurrence in this higher-confidence association set was involvement of mutational signatures that were associated with DNA repair failures in previous analyses of tumor genomes. This included: MMR failures (various SBS; listed in Fig. 5a, b), BER failures (SBS36/56L^15,63,64^, SBS30L/7bL/11L^14,65^), likely NER failures (SBS8L/4L) and replicative DNA polymerase failures (particularly SBS14 and SBS20; additionally SBS56/10aL/36L may be in this group). As tentatively DNA repair-associated signatures, here we additionally considered^6,9,66^ SBS18/36L based on the similarity of the spectrum to the SBS36/18 and because it was found in *MUTYH*-variant patients^63,67^, and additionally SBS33L, SBS54, and SBS-CL1 because they have prominent indel components and were associated with sensitivity to *WRN* loss (Supplementary Fig. 11). Those DNA repair-associated signatures encompass 278 of 701 associations involving mutational signatures in this high-priority set; some individual examples are discussed below. Therefore, mutational signatures resulting from DNA repair failures often result in drug vulnerabilities.

When inspecting the overall balance of sensitivity versus resistance associations, we noted that driver mutations and CNA present a mix of sensitizing and resistance associations. These may be unevenly distributed across genes: see *NRAS* example mentioned above, biased towards resistance, while *EGFR* is biased towards sensitivity (perhaps because mutant EGFR is a target for many approved drugs).

By analogy to this, we also identified two opposing trends in the mutational signatures association tally. Firstly, the signatures associated with DNA repair failures, as listed above, tend to be more often sensitizing (considering relative frequencies of sensitivity to resistance associations shown in Fig. 5c; also see top of the plot in Supplementary Fig. 12a for breakdown by type of test).

Secondly, there is a group of mutational signatures tending towards resistance associations; these signatures also exhibit a higher overall number of associations (Fig. 5c; bottom of plot in Supplementary Fig. 12a). In this group, 6 out of top-7 signatures were previously linked to exposures of mutagenic chemicals: SBS25L (unspecified chemotherapy), SBS18/36L (reactive oxygen species), SBS42L (haloalkane exposure^68^), SBS11 (a DNA methylating agent, the drug TMZ), SBS22L (an agent generating bulky DNA adducts, aristolochic acid^69^), and SBS4/45L (mix of agents from tobacco smoke, where the mutagenesis likely results mainly from DNA adducts by polycyclic aromatic hydrocarbons). Of note, among these, the oxidative damage SBS18/36L does show some sensitizing associations as well (Fig. 5c). These six signatures of mutagen exposures associated with resistance to various drugs, which are overall enriched in drugs targeting e.g. chromatin histone modification, DNA replication, the p53 pathway, JNK and p38 signaling, and ABL signaling (Fig. 5d and Supplementary Fig. 15 breakdown per signature). This data suggests that, overall, mutational signatures of prior chemical exposures in cancer cells commonly predict resistance to future drug exposures.

### Mutational signatures associated with various DNA repair failures predict drug sensitivity

**Fig. 6:**
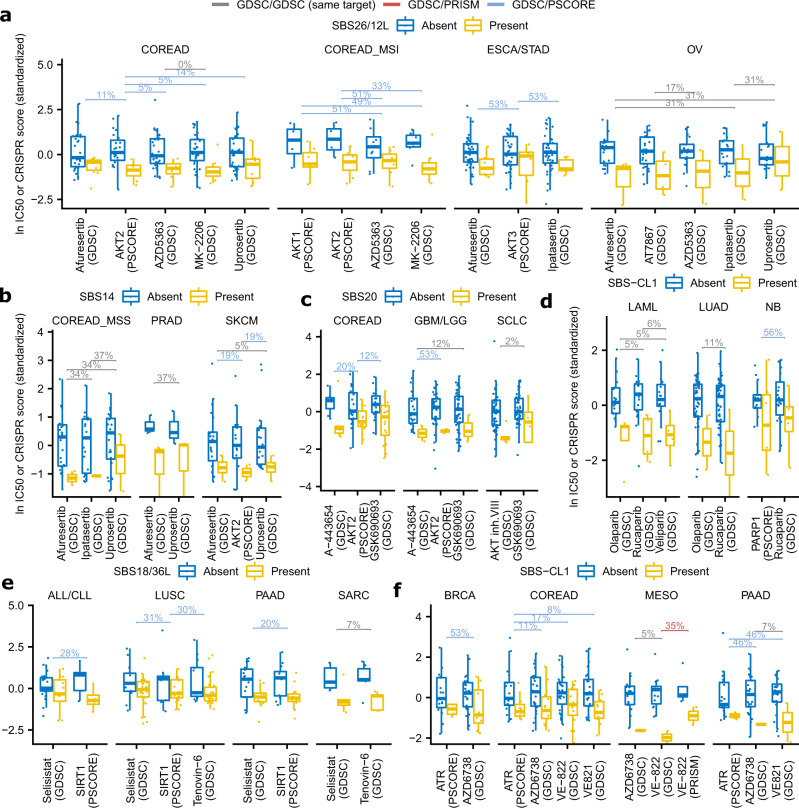
Associations with drug sensitivity or resistance that replicate in independent datasets. **a**–**f** Examples of associations of mutational signatures with drug sensitivity that replicated (using tests in Fig. 3a) multiple times, across different cancer types and/or different types of replication tests. Y-axes show a Z-score derived from either the ln IC50 value (drug sensitivity: “GDSC” or “PRISM” columns) or from the CRISPR essentiality score (“PSCORE” columns). Horizontal brackets show FDR for replicated associations with the presence/absence of a given mutational signature, obtained via a randomization test (Fig. 3a), where color denotes the type of the test (see legend at top of plot). The center lines of box plots denote medians and the box hinges correspond to the 1st and 3rd quartiles, while whiskers extend to 1.5 × IQR from the hinges. Source data are provided as a Source Data file.

A manual curation of the sets of robust associations (Supplementary Data S8) reveals that several MMR signatures associate with sensitivity to AKT serine/threonine kinase inhibitors (Fig. 5a, b). This is seen consistently across many tissues: in colorectal, skin, lung (small cell), and brain (associations at FDR<15%), and additionally in prostate, ovary, and stomach/esophagus cancers (associations at permissive FDR thresholds, all with *p* < 0.005); see Fig. 6a–c for examples involving SBS26/12L, SBS14, and SBS20, respectively. The associations involve 9 different AKTi drugs including uprosertib, MK-2206, and ipatasertib. Several of these drugs have undergone clinical trials showing varying outcomes in unselected patients^70,71^, highlighting the need for identifying predictive biomarkers of response to AKT inhibitors. We considered the possibility that different MMR signatures have varied utility as AKTi markers; indeed, the MMR signature SBS26/12L most commonly associated with sensitivity across different AKTi drugs, with a lower utility of other signatures (Fig. 5a, b). These associations between MMR signatures and AKTi sensitivity may be mechanistically related to associations between *ARID1A* mutations and AKTi sensitivity that we described above (Fig. 3c). Such a link would be consistent with a reported loss of MMR activity in *ARID1A*-mutant cells^72^, and with correlations between ARID1A loss in tumors and MMR deficiencies reported in multiple cancer types^73–75^.

In addition to MMR, signatures resulting from failures in other DNA repair pathways may yield sensitivity associations (Fig. 6d–f and Supplementary Fig. 14). An example is the signature SBS36/56L, possibly indicating failed BER since SBS36 was previously associated with loss-of-function in *MUTYH*. In five cancer types, SBS36 was associated with sensitivity to inhibition of EGFR or of ERBB2 via e.g. afatinib or AST-1306 drugs. These associations were additionally supported in CRISPR k.o. of the *EGFR* or *ERBB2* genes in skin, liver, and head-and-neck cancer (Supplementary Fig. 14c). The related signature SBS18/36L also predicts sensitivity to these agents in three of the five cancer types (Supplementary Fig. 14d). Overall statistical support for sensitivity associations with SBS36/56L and SBS18/36L was higher for the EGFR-targeting drugs than for all other classes of drugs in these cancer types (Supplementary Fig. 14e). Prior studies suggested that EGFR activity can control various DNA repair mechanisms^76–78^.

We further highlight an example involving a signature SBS18/36L, associated with DNA damage by reactive oxygen species, and possibly also with certain deficiencies in the BER pathway due to the similarity of the spectrum with signature 36. This signature was associated with sensitivity to two inhibitors of sirtuin (SIRT) proteins, selisistat and tenovin-6, in pancreatic adenocarcinoma, lung squamous cell carcinoma, sarcoma, and lymphoid leukemia (all tissues at FDRs  ≤ 30%; Fig. 6e). In three out of four tissues the associations with SIRTi replicated in the CRISPR k.o. phenotype of the *SIRT1* gene (Fig. 6e). This adds confidence that SIRT1 may be a promising vulnerability of tumor cells that are undergoing and/or have previously undergone oxidative damage to their DNA and/or have lowered ability to repair such damage (the current analysis does not distinguish between these scenarios).

Another example of a sensitizing mutational signature was interesting due to its occurrence across multiple tissues. The SBS30L/7bL/11L, which is ambiguous but possibly linked to base excision repair failures (since the SBS30 was previously associated with *NTHL1* loss-of-function^14,65^), associates with sensitivity to two related classes of drugs (Supplementary Fig. 14a, b) that converge onto the cytoskeleton. Firstly, there are inhibitors of Aurora kinase A, a protein regulating mitotic spindle assembly and stability, including the drugs ZM447439, Genentech Cpd10, and GSK1070916 (an Aurora B/C inhibitor with some Aurora A activity). These associations are also replicated in the k.o. of the *AURKA* gene (Supplementary Fig. 14a). Secondly, this signature SBS30L/7bL/11L associates with sensitivity to the vinca alkaloids vinblastine, vincristine, and vinorelbine that interfere with assembly of microtubules and forming of the mitotic spindle (Supplementary Fig. 14b). These associations were observed across AML and CML leukemia and liver cancer at FDR ≤ 30%, as well as in colorectal cancer and multiple myeloma at more permissive FDR thresholds (with notable effect sizes, however; Supplementary Fig. 14a, b).

Some associations were found involving mutational signatures recovered from cell line genomes that did not closely match a known SBS spectrum from tumor genomes. An interesting example are associations of the SBS-CL1, an indel-rich signature seen in various cancer types (Supplementary Figs. 2 and 3). Our analysis suggests this associates with sensitivity to DNA damage signaling drugs. Firstly, we identified associations of exposure of SBS-CL1 with sensitivity toward PARP inhibitors olaparib, rucaparib, veliparib, and *PARP1* gene k.o., observed across three cancer types (Fig. 6d). Secondly, we identified associations with sensitivity to ATR inhibitors AZD6738, VE-822, VE-821 or k.o. of the *ATR* gene in four cancer types (Fig. 6f). This example suggests the utility of indel signatures in predicting drug response, in this case to a category of DNA repair drugs that are trialed in the clinic, the ATRi.

Additional associations were recurrently observed across multiple cancer types. Some examples highlighted by a manual curation of these ‘golden set’ associations include: the SBS17aL signature and the ZSTK474 drug and *PIK3CA*/*PIK3CB* genes; SBS3L signature and fedratinib and *JAK2* gene; SBS8L and midostaurin; SBS17b and fludarabine (and possibly more generally DNA antimetabolites). These associations are some representatives among many other examples with a similar degree of confidence (based on the FDRs, and on recurrence across independent tissues/replication tests) in the ‘golden set’ of associations (Supplementary Data S8). In addition to our manual curation, we also provide a prioritization based on a pooled p-value across tissues and different replication tests to highlight the top 10 associations with mutational signatures, and additionally with driver mutations/CNA/DNA hypermethylation, in Supplementary Data S11.

## Discussion

A classical way to treat tumors is to employ DNA damaging drugs and ionizing radiation to target the lessened or overwhelmed capacity for DNA repair in cancer. Since this may manifest as a mutator phenotype, we asked if mutational signatures observed in cancer cells can serve as markers for treatment by drugs or by gene editing. This systematic study generalizes over the known individual examples of mutational patterns stemming from deficient HR^24,26,27^ or MMR, which can guide therapeutic strategies^58–60^.

Cancer cell line panels that were screened for drug response^37,79^ and for gene loss effects^48,80^ provided a resource to test our hypothesis. However, the lack of matched healthy tissues means that extracting somatic mutational signatures using existing methods^31,32,42^ is a challenge. Since germline variation is abundant compared to somatic mutations, even slight variations of germline spectra between populations^34,36^ can affect the trinucleotide context mutation tally. We thus subtracted the expected germline spectrum given the ancestry, and further integrated indel features into mutational signature inference from the cell line WES. Future refinements in the methodology, as well as availability of WGS of cancer cell lines, will improve its accuracy. For instance, this will permit a more detailed set of indel descriptors, as applied in the recent tumor WGS mutation signature studies^9^, in contrast to the limited set of only four indel features we were able to apply in our WES study.

Among mutational signatures that we inferred, the number of associations with drug response was comparable to that of other types of commonly used genomic markers. Hundreds of such associations significantly replicated in independent data and across multiple tissues. Thus mutational signatures appear to be similarly robust predictors as driver mutations or CNA markers. We note that the associations we identified could not have resulted from tissue-specific variation in drug response, since tissues were considered individually in the association analyses (the only exception was merging of some cancer types known to be similar genomically, such as esophagus with stomach cancer, and glioma with glioblastoma).

An important caveat to our study is that the discovered associations do not necessarily imply a causal relationship: the mutational signature-generating process and the drug phenotype may be only indirectly associated. In other words, the mutational signature might serve as a marker for another alteration (e.g. a driver mutation), which may be the proximal cause of the drug sensitivity or resistance. This is similarly possible with mutational signatures as with other genetic markers (mutations or CNA in cancer genes), and also with gene expression markers. Because these various sets of genomic/transcriptomic features can correlate across tumors, prioritizing the likely causal relations for further follow-up is a challenge that remains to be addressed; larger cell line panels will be helpful, as well as use of isogenic cell panels.

Furthermore, another issue are false negatives in identifying associations – again similarly so with mutational signatures as with other markers – due to sparse data, where often only few cell lines from a cancer type bear each marker. Thus, statistical power may be limited for many markers in current cell line screens. Additionally, because drug sensitivity measurements can be noisy (as evidenced in less-than-ideal agreement between different screening data sets^43–45^) replication analyses across diverse datasets will be conservatively biased. It bears mentioning that absence of a significant association in our lists does not imply there is not an association of that marker with that drug, but might rather mean that the analysis at current sample sizes may be underpowered to detect the association (see e.g. Supplementary Fig. 13b).

A key question to be addressed in future work is the clinical relevance of the mutational signature drug markers, which we here identified in cancer cell line panel data. Performing association studies on tumor genomic datasets (for which the clinical data about treatments and patient response are sometimes available) is complicated by the diversity of therapeutic regimes: most patients are treated with multiple overlapping sets of drugs and possibly radiotherapy, which makes it challenging to identify effects of individual drugs by retrospective analysis. Additionally, the drug assignment may be confounded by demographics and by cancer stage/grade or subtype, further complicating analysis. Large controlled randomized trials with treatment and control arms, for which the tumor genomic data is also available, would facilitate identifying various types of genomic markers (mutational signatures or otherwise) relevant to drug response and patient survival.

With respect to mechanistic insight, future improvements in methodology will refine the mutation signature markers and clarify the underlying mechanisms. For instance, a known limitation of various mutational signature extraction methods – including ours – is the difficulty of discerning the ‘featureless’ signatures such as SBS3, SBS5, and SBS8^81^. A further issue that merits attention is timing: a genomic analysis of cell lines^31^ suggested that the activity of some mutational processes is variable in time. While a genome sequence reflects a record of mutagenic activity in the past, it may or may not reflect current mutagenic activity, which is presumably more relevant for drug sensitivity phenotypes. Occurrence of recently active signatures is difficult to identify from bulk DNA sequencing from cell culture, as the recent mutations may not rise to sufficient allele frequencies to be detected, and may conservatively bias the results of an association analysis such as ours. We note that a related issue could affect also the more established markers i.e. driver mutations or CNAs in cancer genes: given the rapid accumulation of genetic changes in cultured cancer cell lines^82,83^, and prevalent epistasis in cancer^84,85^, it is plausible that recently occurring, unobserved mutations or CNAs affect the ability to identify drug sensitivity markers from analyses of cell line screening data.

An interesting observation about mutational signatures associated with drug activity is that some likely result not from DNA repair deficiencies, but instead from exposure to mutagenic agents. Some of these signatures presented many associations in our analyses and were, overall, more commonly associated with drug resistance rather than sensitivity (Supplementary Fig. 12 and Figs. 4 and 6a, b). For example, this includes SBS25 and SBS11, reported to be associated with chemotherapy, or signatures linked with exposure to chemicals causing DNA adducts (tobacco smoking, aristolochic acid), and additionally an SBS17a-like signature as well (where SBS17 was associated with gastric acid exposure, possibly via oxidative damage to the nucleotide pool). Even though cell lines are not exposed to these chemical agents during culture and thus the signatures are presumably not ‘active’, drug associations are identified with such signatures (Fig. 4).

One possible explanation may be that mutational signatures of different processes are sometimes sufficiently similar such that they are not easily ‘unmixed’ only from trinucleotide spectra. For instance, SBS17 (mostly A>C/T>G changes) might result from varied mechanisms that converge onto the same spectrum, some of which are from chemotherapy exposure, while others may be endogenous^86^. This highlights an example where current statistical methods may not reliably deconvolve underlying biological mechanisms. This might be addressed by the use of additional mutational features, such as penta- or hepta-nucleotide contexts^87^, small indels^9^, copy-number changes^22^ and strand-specific, or regional mutation rates^10,88^.

Another explanation may be that prior exposure to a carcinogen would select tumor cells with an altered DNA replication/repair state, which continues after the carcinogen is withdrawn, thus generating resistance in cancer cells. Indeed, prolonged exposure to mutagens that are also cytotoxic – as is the case for many cancer therapeutics – is likely to select resistant cells, in some cases via altered DNA replication or repair mechanisms. For instance, therapy of tumors with temozolomide (associated with SBS11) is known to select for cells that are resistant due to a MMR-deficiency via loss-of-function of *MSH6*^89^. It is conceivable that also the various epigenetic changes resulting from carcinogen exposure might confer similar properties. In other words, even a temporary exposure to a mutagen may prime tumor cells for resisting later drug treatment.

## Methods

### Human cancer cell lines and primary tumor data

We obtained WES bam files (human reference genome version hg19) of human cancer cell lines (*n* = 1072; cancer cell lines from Genomics in Drug Sensitivity in Cancer (GDSC)) from European Genome-phenome Archive (EGA) (ID number: EGAD00001001039) and WES bam files (human reference genome version hg38) of primary tumors (*n* = 6154) and their matched normal samples (*n* = 6154) from the TCGA repository at NCI Genomic Data Commons (access via dbGaP accession phs000178). We downloaded samples from the following TCGA cohorts: BLCA, BRCA, COAD, GBM, HNSC, KICH, KIRC, KIRP, LIHC, LUAD, LUSC, OV, PAAD, READ, STAD, THCA, UCEC. We aligned the human cancer cell line bam files to the human reference genome (version hg38) using the bwa^90^ software, and sorted and indexed them using the *samtools* software^91^. We used the Strelka2 software (version 2.8.4)^92^ to call single nucleotide variants (SNVs) and small insertions and deletions. We called SNVs and indels for cell lines, primary tumors and normal samples. In samples where Strelka2 was unable to run, a re-alignment was performed using Picard tools (version 2.18.7)^93^ to convert the bams to FASTQ and, following that, the alignment was performed by executing bwa sampe (version 0.7.16a) with default parameters. The resulting bam files were sorted and indexed using Picard tools. We used SNVs and indels marked as “PASS” in the Strelka2 output. We annotated SNVs and indels with minor allele frequencies (MAF) obtained from the gnomAD database^94^ (for SNVs and indels that could be found in gnomAD).

### Data for human cancer cell lines

We downloaded drug response data for 518 drugs from GDSC (Release 8.3; June 2019)^37^. We used the natural logarithm of the 50% growth inhibition values (IC50) as a measure of the activity of a compound against a given cell line. If for the same drug activities were available from both GDSC1 and GDSC2 versions, we used GDSC1. We downloaded the information about drugs (the list of drugs’ putative targets and target pathways) from the GDSC website (https://www.cancerrxgene.org/). We manually curated the list to correct inconsistencies (Supplementary Data S12).

We obtained the following genetic features of cancer cell lines from the GDSC repository:^30^ cancer driver genes (Muts), regions of recurrent focal copy number alterations (CNAs), hypermethylated informative CpG islands (HypMet), and microsatellite instability status (MSI). We downloaded ANOVA_input.txt files for 18 cancer types and pan-cancer analysis from https://www.cancerrxgene.org/gdsc1000/GDSC1000_WebResources/Pharmacogenomic_interactions.html. We downloaded cancer cell line gene expression data (“sanger1018_brainarray_ensemblgene_rma.txt”) from https://www.cancerrxgene.org/. We selected expression data for 1564 genes corresponding to the L1000 assay^39^ and to known drug targets^40^.

The drug response data (IC50) for 1502 drugs was downloaded from the PRISM^46^ database (secondary-screen-dose-response-curve-parameters.csv). Drugs and cell lines from PRISM and GDSC databases were matched via drug names, obtaining in total 348 cell lines and 178 drugs that overlap between the two databases.

The gene-level fitness effects for 16,827 genes in 786 cancer cell lines were downloaded as the Integrated cancer dependency dataset from Wellcome Sanger Institute (release 1) and Broad Institute (19Q3)

(“integrated_Sanger_Broad_essentiality_matrices_20200402.zip”) from the Project SCORE database^48^ and matched to the GDSC cell lines via cell line names, obtaining in total 517 overlapping cell lines (https://score.depmap.sanger.ac.uk/downloads).

### Matching of cancer cell lines and primary tumors (ancestry matching procedure)

To ensure that genomic data is comparable between cell lines and tumors we performed the following steps: (i) we used only SNVs detected in the regions of the exome with well sequencing coverage (≥20 reads in 9 out of 10 randomly selected samples); (ii) to avoid gender bias during analysis, variants on X and Y chromosomes were not used; (iii) only uniquely mappable regions of the genome were used, as defined by the Umap k36 criterion;^95^ (iv) we discarded regions with frequent somatic deletions, namely pan-cancer deletions of significant SCNA^96^ and frequently deleted chromosome arms (deleted in >18% of tumor samples);^97^ (v) we detected copy number changes of cell lines with CNVkit^98^ and removed deleted regions (log2 score < −0.3). From the remaining regions of the exome we selected common germline variants across the cell line exomes and the TCGA exomes (MAF>5% in GnomAD).To perform ‘ancestry matching’ of cancer cell lines with TCGA normal samples, we performed Principal Component Analysis (PCA) on the matrix of common germline variants, followed by clustering according to principal components (PC) (see below).

### Clustering of cell lines and TCGA germline samples

We employed robust clustering (“tclust” algorithm;^99^ discards outlying samples) on the first 140 principal components derived from the common germline variants. Initially, we considered the first 150 principal components, however, some PCs were attributed to the batch effect, i.e., they separate cell lines from TCGA samples. We removed the top 10 such PCs as determined by feature importance of the random forest classifier (“randomForestSRC” R package) trained to distinguish between TCGA samples and cell lines. We used the remaining 140 PCs as an input to the tclust algorithm. Outlying samples, as determined by tclust, were discarded. We varied the number of clusters from 4 to 20, determining the optimal number of clusters (13) by using simulated cell line exomes (Fig. 1c; see below). The cancer cell lines were matched to their ‘ancestry-matched’ TCGA normal samples, i.e., those belonging to the same cluster as the cell line. We then used ‘ancestors’ to augment the filtering of germline SNVs from cancer cell line exomes, in addition to filtering according to the common practices to retain only variants absent or present in very low frequencies in population databases (see below).

### Filtering of germline SNVs from cell lines

Here we considered SNVs from the regions of the exome as specified before (steps i–v), with the difference that we used SNVs with the sequencing coverage of ≥8 reads in at least 90% samples (9 out of 10 randomly selected samples). We next filtered germline variants from cancer cell lines following common practices^29,31,79^ – here, removing population variants (found at MAF>0.001% in the gnomAD population database); additionally, we filtered germline variants that appeared >5% of samples in our TCGA data set or cell lines data set which removes germline variants that might be particular to this data set and also suspected sequencing artifacts). Next, from the remaining SNVs, for each cell line and TCGA germline sample, we calculated its trinucleotide mutation spectrum. The mutation spectrum contains 96 components, which are the counts of six possible mutation types (C>T, C>A, C>G, A>T, A>G, and A>C, considered DNA strand-symmetrically) in 16 possible 5′ and 3′ neighboring nucleotide contexts for each mutation type. For each cell line, from the cell line’s 96 trinucleotide spectrum, we subtracted the median 96 trinucleotide frequencies of its TCGA ‘ancestry-matched samples’ (i.e. the TCGA normal samples belonging to the same population cluster as the cell line in the PC analysis of common germline variants, see above). In case the subtraction resulted in a negative count for some of the contexts, we set it to zero.

### Insertion and deletion types

In addition to the standard 96 trinucleotide spectra, to extract mutational signatures from cell lines (see below) we considered additional features based on small insertions and deletions. We filtered the regions of the exome using the steps (i-iv) as described above. Similarly as for SNVs, we discarded indels found at MAF>0.001% in the gnomAD population database and filtered the ones that appeared in >5% of cell line samples. The remaining indels were classified into 4, 5, 8, 14, or 32 different indel types considering: the length of insertion or deletion (considering lengths 1, 2, 3–4, 5+), microhomology at deletion sites (considering the lengths of microhomology 1 or 2+), and microsatellites at indel loci (considering repeat sizes of 1 and 2–5+). We benchmarked the cell line mutational signatures (see below) that used different indel types. The most favorable indel types according to that benchmark were the simplest where we differentiate 4 indel types: deletions with microhomology (Del-MH), deletions at microsatellite loci (Del-MS), other deletions (Del-Other), and insertions at any locus (Insertion).

### Simulated cell line exomes

We used TCGA exomes to simulate cell line exomes (or more precisely, the variant calls that would originate from a cell line exome) in order to benchmark our ancestry matching procedure for efficacy in reconstructing the mutation spectrum of cancer cell line exomes. Additionally, we used the benchmark to optimize the number of clusters in the inference of subpopulations. To simulate variant calls from cell line exomes, we performed the variant calling for tumor samples in the same way as for the cell lines, i.e., without matched normal samples. In addition, for every sample we merged SNV calls obtained thus with the somatic SNV calls of tumors, ensuring that the true somatic mutations are a part of a simulated cell line to enable an accurate estimation of errors. In the tumor types that were represented both in the set of cancer cell lines and the set of TCGA tumors, we randomly selected 450 tumor samples taking approximately the same number of samples per tumor type as in cell lines. In the subsequent analysis involving simulated cell line exomes, we removed their corresponding normal samples from the pool of germline samples. We performed the ancestry matching procedure involving simulated cell line exomes in the same way as described above (Fig. 1g) and evaluated the accuracy of mutation spectrum reconstruction by comparing the mutation spectrum of a tumor (ground truth) to the corresponding simulated cell line mutation spectrum obtained by ancestry matching. As an accuracy measure, we used Absolute Error (the sum of absolute values of differences between the 96 components of the ground truth spectrum and the reconstructed spectrum). To estimate the optimal number of subpopulations we varied the number of clusters in the tclust algorithm from 2 to 20, with 13 being the optimal according to the average Absolute Error of 450 simulated cell lines (Fig. 1c).

To investigate the variation between germline mutational spectra across human (sub)populations (as reported recently^34^), we performed hierarchical clustering (based on cosine similarity; stats package in R) of the median mutation spectra of TCGA subpopulations (i.e., median mutation spectra of the TCGA normal samples within clusters) (Supplementary Fig. 1E). In addition, we performed PC analysis of mutation spectra of TCGA normal samples showing that the main principal component separates the main ethnicity groups (Supplementary Fig. 1D). We note that, in the previous steps, clustering to infer subpopulations was performed via common variants (see above), and the trinucleotide spectra were determined after excluding the non-rare variants (MAF >0.001% in the gnomAD population database and variants that appear in >5% of samples in our TCGA data set) ruling out circularity.

Next, we compared our ancestry matching procedure (involving 13 clusters) to the baseline method of determining somatic variants in a cell line genome (i.e., filtering of germline variants according to MAF, where SNVs with MAF >0.001% and that appeared in >5% of samples in our dataset are removed) and the bootstrap self-similarity method (Fig. 1d). The bootstrap self-similarity error was calculated as an error between true and randomly perturbed somatic mutation spectrum, averaged over 100 random runs. For random perturbation, we used the “sampling” function of the “UPmultinomial” R package.

We compared the true number of somatic mutations versus the number obtained with the ancestry-matching and the MAF filtering (removal of SNVs with MAF where MAF>0.001% and that appeared in >5% of samples) to quantify the degree of overfiltering and underfiltering of these two filtering approaches (Supplementary Fig. 1h). If after filtering, the simulated cell line exome has less mutations than the number of true somatic mutations, we consider it overfiltered (i.e., some of the true somatic mutations were removed). Otherwise, if it contains more mutations than the number of true somatic mutations, we consider it underfiltered (i.e., residual germline variants remained). Next, we compared the accuracy of mutational signature extraction (see below for the method) with the trinucleotide profiles of simulated cell lines obtained with MAF filtering and the ‘ancestry matching’ approach. We compared the 20 known mutational signatures (>0.85 cosine similarity to PCAWG signatures) that were extracted from both sets of trinucleotide profiles to the mutational signatures extracted from the true somatic trinucleotide profiles. We compared the cosine similarity of trinucleotide composition of the signatures and the cosine similarity of the exposure scores. The ‘ancestry matching’ signatures show statistically significant closer match to the true somatic signatures than ‘MAF filtering’ signatures in both trinucleotide composition and exposure scores (Supplementary Fig. 1g). *P*-values were calculated by Wilcoxon rank-sum test (artifact signatures were excluded from the test).

### Extraction of mutational signatures

We extracted cancer cell line mutational signatures from 96 component trinucleotide mutation spectra of cancer cell lines obtained with the ancestry matching procedure. In total, we used mutation spectra of 930 cancer cell line exomes (Supplementary Data S13). Note that some cell lines were excluded because they were assigned to the outlier cluster of the tclust algorithm, based on their common germline variants (see above). To extract mutational signatures we used a custom R script based on non-negative matrix factorization (NMF) as described by Alexandrov et al.^33^. We additionally implemented a number of signature extraction procedures proposed in the literature recently^9,38,42^ and benchmarked the resulting signatures according to the several criteria: (a) The agreement with an established set of SBS signatures (PCAWG signatures^9^) measured as the number of PCAWG signatures that were recapitulated at ≥0.85 cosine similarity cutoff; (b) The similarity of the cell line signature exposure profiles across cancer types to the exposure profiles of PCAWG signatures, measured as cosine similarity between an average exposure per-cancer-type profile of a cell line signature and its matching PCAWG signature (for signatures recapitulated at ≥0.85 cosine similarity); (c) The accuracy of the signatures in predicting drug sensitivity profiles across cancer cell line panels (see below for method).

From the matrix containing mutation spectra of samples, we generated 300 matrices by bootstrap resampling. One bootstrap sample is obtained by applying the sampling function of the “UPmultinomial” R package to each sample’s spectrum. Next, we applied the NMF algorithm to each of the bootstrap samples (“nmf” function of the “nmfgpu4R” R package to obtain different NMF runs; we used the Multiplicative update rules algorithm^100^ with 10,000 as the maximal number of iterations). For each bootstrap sample, we varied the number of signatures from 2 to 40. Computations were performed on an NVIDIA GeForce RTX 2080 Ti GPU.

We implemented the following modifications to the above basic procedure (each tested independently and some in combinations; Supplementary Data S1) to obtain the candidate cell line signatures which were matched to the known tumor PCAWG signatures (see below): (i) Similarly as done in the seminal work of Alexandrov et al.^33^, we clustered each batch of NMF solutions and used the cluster medoids as candidate cell line signatures. We used the clara function in clusters R package, with Euclidean distance and standardization and “pamLike” options; the number of samples to be drawn from the dataset was set to 10% of the number of samples. Each batch of NMF solutions obtained as described before (one batch corresponds to 300 x n solutions, where *n* is the number of signatures; varies from 2 to 40) was clustered into k clusters with k-means clustering, varying k from 2 to 30. (ii) We applied the RTOL-filtering (relative tolerance) as proposed by Degasperi et al.^38^, where (separately for each different number of signatures parameter) NMF runs that diverge for more than 0.1% from the best NMF run are removed, as measured by the root-mean-square deviation of the factorization. (iii) We implemented the ‘hierarchical extraction’ procedure proposed by Alexandrov et al.^9^ where NMF is repeated iteratively while removing (or down weighting) the well-reconstructed samples (cosine similarity above a specified threshold) from a previous iteration to uncover additional signatures. We allowed a maximum of 3 iterations and tested different cosine similarity thresholds ranging from 0.95 to 0.99. In the case of down weighting, we multiplied the sample’s mutation spectra by 0.05 instead of removing it. (iv) Similarly as in Ghandi et al.^42^, we performed joint signatures inference of cell line exomes and tumor exomes. To the initial matrix containing mutation spectra of cell lines, we added mutation spectra of 6154 TCGA somatic exomes which were preprocessed in the same way as cell line exomes: the same regions of the exome were used (see above for filtering by coverage, mappability, etc.).

From the candidate cancer cell line mutational signatures obtained by the above described methodologies, we searched for the signatures that closely resemble the ones that were previously found in human cancers^9^ (referred to as PCAWG signatures in the text). We compared the individual signatures to the PCAWG signatures searching for the closest matching cell line signature for each PCAWG signature. As a final set of tumor-like cell line signatures, we kept the closest matching cell line signature if its cosine similarity to the best matching PCAWG signature exceeded 0.85. For cell line signatures that used indel features in addition to 96 tri-nucleotide spectra, cosine similarity was calculated only on 96 spectra since the known PCAWG SBS mutational signatures do not have an indel component in their current implementation (v3.2). We considered an additional criterion for matching cell line and tumor signatures where in addition to the mutation-spectrum cosine similarity, a cosine similarity between signature’s average per-cancer-type exposure profile (for cancer types available in both cell line and PCAWG data). The spectrum-cosine and exposure-cosine similarities were combined into a single metric given the weight ‘*w*’ controlling the relative contribution of each: *w*×spectrum-cosine + (1–*w*) × exposure-cosine. We tested different weights: *w* = 0.5, 0.7, 0.9, and 0.99. For each PCAWG signature, from a set of cell line signatures that match it with spectrum-cosine ≥0.85, we selected the best matching cell line signature as the one with the highest combined metric.

We considered using additional regression post-processing for assigning signature exposures to samples, similarly as done by Petljak et al.^31^ and Alexandrov et al.^9^. We used the “sigproSS” tool of SigProfilerExtractor framework^101^ to attribute exposures to the extracted cell line signatures (i.e., signatures were used as an input to sigproSS). Additionally, we used a custom script based on regularized regression from “glmnet” R package enforcing different degrees of sparseness. We considered Ridge, Lasso and Elastic Net regression to model cell line’s mutation spectra as a combination of cell line signatures, where the resulting regression coefficients are considered as exposures to signatures. We enforced non-negative coefficients, no interaction term, and used crossvalidation to determine the optimal value of the lambda parameter.

The different procedures for mutational signature extraction we implemented were evaluated according to the above-described criteria (a–c), results are presented in Supplementary Data S1. As a final set of signatures we chose signatures obtained with hierarchical extraction (using 0.97 cosine similarity threshold for sample removal), using 4 indel features in addition to 96 trinucleotide types. We considered both the cosine similarity in the trinucleotide spectrum and also the cosine similarity in the exposures across tissues (in tumor genomes), using *w* = 0.9 as weight on the trinucleotide-cosine and 0.1 on exposure-cosine, for matching our cell line signatures with the known COSMIC tumor signatures. No post-processing of exposures to signatures was performed since it did not yield improvements according to the evaluation criteria, thus, we used the raw NMF scores of the exposure matrix. A notable modification from the signature extraction method presented in Alexandrov et al.^33^ is that we did not limit to a single value of the “number of signatures” NMF parameter (choosing based on measures of fit and consistency, as in Alexandrov et al.^33^). Instead, inference was run for many values of this parameter, and the final set of mutational signatures consists of solutions from different values for the ‘number of signatures’ parameter.

This procedure yielded 52 signatures (Supplementary Fig. 2). The cell line signatures are named according to the PCAWG signatures they resemble, e.g., the cell line signature name SBS4/45L denotes that for PCAWG SBS4 this signature was the closest match (i.e., SBS4 is the primary signature); 45L denotes that the signature also resembled PCAWG signature SBS45 (at cosine similarity ≥0.85). The suffix “L” (for “like”) denotes 0.85 ≤ cosine similarity < 0.95 (a somewhat less-close match), while the absence of the suffix “L” means cosine similarity ≥0.95. Names of signatures other than the primary signature (if present) are ordered by decreasing cosine similarity.

We checked if the trinucleotide composition of the exome ‘territory’ covered in WES sequencing we used could affect the cosine similarities to the known PCAWG signatures, since they were extracted from the WGS data. We adjusted the trinucleotide spectra of our signatures to match the WGS spectra and re-calculated cosine similarities to the PCAWG signatures. The cosine similarities are not substantially affected – our signatures map to PCAWG signatures largely irrespective of the adjustment for territories (Supplementary Fig. 17b). Note the one signature that does change between the two normalizations (0.96 vs 0.87) is an SBS49-like signature, where SBS49 was previously suggested to be an artifact^9^. Furthermore, to check that the mutational signatures extraction was not biased in terms of the trinucleotide composition due to the removal of lowly covered regions and the common germline loci, we compared the trinucleotide compositions in an exome, our examined territory (largely the exome with lowly-covered regions removed), and in our examined territory after having removed common SNP loci. The trinucleotide composition was highly similar (cosine similarities >0.993 in all three possible pairwise comparisons; Supplementary Fig. 17a).

We next searched for cell line-specific signatures, i.e., signatures that commonly appear in cell line data and do not resemble any of the known tumor signatures. To this end, we employed k-means clustering (“clara” function in “clusters” R package, with Euclidean distance, standardization, “pamLike” options and 10% as the number of samples to be drawn from the dataset). Each batch of NMF solutions (from the signature extraction method selected as final by the evaluation) was clustered into *k* clusters with k-means clustering varying *k* from 2 to 40. We chose the clustering result (i.e., a set of signatures) where the agreement with PCAWG signatures was maximized in terms of the number of cluster medoids that resemble PCAWG signatures (at cosine similarity ≥0.85). From such a set of signatures we selected the ones dissimilar from any of PCAWG signatures (cosine similarity <0.8), yielding in total 5 cancer cell line-specific signatures (named SBS-CL). These SBS-CL signatures appear together with real signatures and are robust (i.e., they are cluster medoids) therefore they are also likely bona fide mutational signatures that might originate, for instance, from cell-line specific mutational processes.

In total, this yielded 57 cancer cell line signatures (52 corresponding to known tumor signatures, and 5 additional cell line-specific signatures) (Supplementary Fig. 2). To investigate if some of the extracted signatures are a result of incomplete separation of other signatures we considered the coefficients of the Lasso regression (“glmnet” R package) (Supplementary Fig. 6), where we modeled cell line signatures extracted in this work as a linear combination of PCAWG signatures (Supplementary Fig. 6).

### Predicting drug response

We built predictive models of drug response using the Random Forest (RF) algorithm as implemented in the “randomForestSRC” R package. We compared the predictive performance of different predictors: mutations in cancer driver genes (Muts), recurrent copy number alterations (CNAs), DNA hypermethylation (HypMet), gene expression, previously reported cancer cell line mutational signatures^31,32,42^, and mutational signatures extracted in this work. We used Muts, CNAs, and HypMet data as reported by Iorio et al.^30^, namely, mutations in cancer genes associated with positive selection in tumors, focal recurrently aberrant copy number segments, and hypermethylated informative 5′C-phosphate-G-3′ sites in gene promoters. The dependent variable was the continuous value of a response to a drug (log IC50); we iterated this over all drugs. Another possible way to run Random Forest, not employed here, would be to binarize the log IC50 drug response and run the RF algorithm in classification mode rather than regression mode. However, binarizing the drug response implies some loss of information, and moreover, it would necessitate an extra parameter in the analysis (choice of the binarization threshold) so we opted to use continuous drug response values here.

We built cancer type specific models for each drug separately, considering only models where at least 15 cell lines with drug response were available to train the model. For model validation we used 10-fold cross validation which was repeated five times to get more stable results. We built random forests with 100 trees and a minimal number of samples in terminal nodes set to 2. We assessed the predictive performance of the predictors by the relative root-mean-square-error (RRMSE), i.e., a root-mean-square error divided by the root-mean-square error of the default model. The default model predicts a constant value for all cell lines equal to the average ln IC50 across the training set. RRMSE < 1, therefore, denotes better accuracy better than the one of the (uninformative) default model. We define such models as predictive. Results are presented as an average RRMSE per cancer type across all models built for a cancer type (Fig. 2a). Note that, all drugs were not necessarily tested exhaustively across all cancer cell lines, therefore, the number of models per cancer type may differ (one model corresponds to one drug) due to missing data (<15 cell lines with drug response data available), as well as the number of model per different predictors (mutational signatures, gene expression, oncogenic mutations, copy number alterations, DNA hypermethylation) due to data availability. In the analysis of complementarity between different predictor types (Fig. 2d, e and Supplementary Fig. 7b) we thus report relative numbers of predictive models to facilitate fair comparison.

To assess the statistical significance of the differences in the predictive performance (Fig. 2b, c), we follow the recommendations given by Demšar^102^. More specifically, to statistically compare the predictive performance of multiple predictors over multiple datasets we use the corrected Friedman test and the post-hoc Nemenyi test^103^. Here, a dataset corresponds to a pair of one cancer-type and one drug. Due to the requirements of this test, only the intersection of drugs modeled across all cancer types and predictors were considered (due to missing data the number of drugs modeled per cancer type and/or predictor may differ; see above). For each drug-cancer-type pair, predictors are ranked according to their RRMSE for that drug-cancer-type pair, where rank 1 corresponds to the best (i.e. the lowest) RRMSE, 2 to second-best, etc. Ranks are then averaged across all drug-cancer-type pairs to obtain the average rankings of predictors. The Nemenyi test performs a pairwise comparison of predictors’ performance based on the absolute difference of the average rankings of the predictors to assess whether there are statistically significant differences among predictors. The test determines the critical difference (CD) for a given significance level α, if the difference between the average ranks of two predictors is greater than CD, the null hypothesis (that the predictors have the same performance) is rejected., i.e., there is a statistically significant difference between the two predictors. The results from the Nemenyi post-hoc test are presented with an average ranks diagram^102^. The average predictor rankings are depicted on an axis, in such a manner that the best ranking algorithms are at the left-most side of the diagram. The algorithms that do not differ significantly (in performance) for a significance level of 0.05 are connected with a bold line, therefore, predictors that are not connected are statistically significantly different according to the test.

### Associations with drug response

#### Randomization test for associations that replicate in independent datasets

We performed two-way association testing where we searched for robust associations that replicate in two independent datasets (schematic in Fig. 3a). We considered three different types of two-way tests where we enforced that, for a given drug, an association between a particular feature (a mutational signature or a genetic feature) and the drug response from the GDSC database is replicated: (1) in the PRISM drug screening database (GDSC/PRISM test) for the same drug, or (2) with another drug from the GDSC database that shares the same molecular target (GDSC/GDSC (same target) test), or (3) in the Project SCORE CRISPR/Cas9 genetic screen as an association with a protein-coding gene fitness score of one of the drug’s target proteins (GDSC/PSCORE test).

We considered cancer-type specific associations. We required that in both tests of a two-way test an association is detected in the same cancer type. We merged some similar cancer types with a small number of cell lines: esophagus carcinoma and stomach adenocarcinoma (denoted as ESCA/STAD), glioblastoma and brain lower grade glioma (denoted as GBM/LGG), and head and neck squamous cell carcinoma and lung squamous cell carcinoma (denoted as HNSC/LUSC). We additionally considered two groups of cell lines obtained by dividing colorectal cell lines according to microsatellite instability status (denoted as COREAD_MSI and COREAD_MSS; other cancer types did not have enough cell lines with microsatellite (in)stability labels to warrant such division). Prior to the association search, we removed 21 cell lines that exhibited either sensitivity or resistance nonspecifically towards a large number of drugs (15 cell lines reported by Abbas-Aghababazadeh et al.^104^ and 6 outlier cell lines considering median ln IC50), as well as additional 29 cell lines that were reported to be misclassified^105^. In addition, for each cancer type, we removed outlier cell lines by the total number of mutations (remaining after the filtering with the ‘ancestry matching’ procedure; see above), here defined as having the number of mutations >3× interquartile range + upper quartile, or <3 × interquartile range - lower quartile (calculated for each cancer type separately). We used binarized exposures to mutational signatures where, for each signature, values below the 5% of the value of the second-highest exposure score (used in order to avoid single high-score outliers observed in some signatures) of that signature were set to 0, and the rest to 1 (Supplementary Data S4). We empirically tested several different thresholds ranging from 1% to 20% (of the second-highest exposure across cell lines), and measured (i) the sparsity of binarized signatures and (ii) the distribution of binarized exposures across tissues for signatures with known etiology that should dominantly appear in certain tissues (the “UV” signatures 7a and 7b in skin and the “tobacco” signature 4 in lung). We chose 5% since it offered a good tradeoff on these two metrics; sparsity is not too high, while the tissue distribution of signatures 7a, 7b, and 4 are reasonable (Supplementary Fig. 15B). We used normalized (relative) signature exposures, as described above. We considered only tests with at least 8 cancer cell lines and at least 2 non-zero values of a feature.

All of the association tests were performed by modeling the drug response (or gene fitness score) to associate it with the status of a feature (i.e., a mutational signature or a genetic feature) searching separately for sensitivity and resistance associations. For each association, we calculated the association score as the minimum (in the case of sensitivity) or maximum (resistance) effect size (Cohen’s *d*) of the two independent datasets i.e. positive Cohen’s *d* implies sensitivity and negative resistance associations. Here, effect size is the Cohen’s *d* statistic: difference of mean drug sensitivity (ln IC50) between the cell lines having the feature and those not having it, divided by the pooled standard deviation of the data. To obtain the association’s empirical *p*-value, we performed a randomization procedure where we calculated the association score 100,000 times for the randomly shuffled features. For the sensitivity test, the formula for the *p* value is: $p=\frac{{{{{{\rm{random}}}}}}\,{{{{{\rm{score}}}}}}\, > =\,{{{{{\rm{observed}}}}}}\,{{{{{\rm{score}}}}}}}{{{{{{\rm{num}}}}}}.{{{{{\rm{of}}}}}}\,{{{{{\rm{randomizations}}}}}}}$ and for the resistance is: $p=\frac{{{{{{\rm{random}}}}}}\,{{{{{\rm{score}}}}}}\, < =\,{{{{{\rm{observed}}}}}}\,{{{{{\rm{score}}}}}}}{{{{{{\rm{num}}}}}}.\,{{{{{\rm{of}}}}}}\,{{{{{\rm{randomizations}}}}}}}$. Due to the computational burden, we performed the randomization procedure only for associations that had an effect size >0.2 in the primary test. The empirical *p*-values were adjusted with the Tibshirani-Storey method^47^.

Recent work suggested that MMR-failure signatures can be grouped into a few broad types^38,106,107^, in particular a group enriched with C > T changes, and a group enriched with T>C (equivalently, A > G) changes. Based on this we considered aggregated mis-match mutational signatures where we merged p-values and effect sizes of SBS6, SBS15, and SBS44L (denoted as SBS-MMR1); SBS21 and SBS26/12L (denoted as SBS-MMR2). Similarly, we considered the aggregate of the two APOBEC signatures SBS2 and SBS13 (denoted as SBS-APOBEC). Pooled p-values of aggregated signatures were obtained by Fisher's method, while pooled effect size was obtained by averaging. Pooled p-values were adjusted the same as described above.

We consider a two-way association as statistically significant if FDR<15% and additionally we imposed an effect size threshold of Cohen’s *d* > 0.5 in both tests (based on a known set of positive control associations (Supplementary Fig. 16) for GDSC/PRISM and GDSC/PSCORE tests, while for the GDSC/GDSC (same target) test we required Cohen’s *d* > 1. In addition, we considered only associations coming from cancer types where the inflation factor lambda was below 1.3 (Supplementary Fig. 8). Note that, in supplementary data also associations with lambda > 1.3 are listed. For some analyses we considered an additional set of associations with an unadjusted p-value threshold of < 0.005 and the same effect size threshold of Cohen’s *d* > 0.5. As a rule-of-thumb for interpretation, Cohen’s *d* = 0.2, 0.5 and 0.8 correspond to small, medium and large effect sizes, respectively^108^. We used “QCEWAS” R package to calculate the lambda score to estimate the inflation of *p*-values (calculated separately for sensitivity and resistance associations).

#### ‘Golden’ and ‘Silver’ sets of high-priority associations

We collated a list of 3911 associations (the ‘silver set’; Supplementary Data S10). To make this list, we considered all associations tested in the three ‘two-way’ replication tests that pass the permissive criterion of significance (effect size *d* > 0.5 in both tests (or *d* > 1 for the GDSC/GDSC two-way test) and a nominal *p* < 0.005). From these, an association (between a feature and drug within a certain cancer type) was listed in the silver set if it was confirmed in more than one ‘two-way’ replication tests, or was seen in more than one cancer type. We require that at least one of the supporting associations has FDR<25%. Additionally, we collated a ‘golden set’ of high-priority list of associations involving mutational signatures and cancer functional events which we consider to be suitable for follow up work: we require that an association involving the same drug is supported in at least three cancer types or is replicated in all three ‘two-way’ tests where at least one association has FDR < 25% (995 associations; Supplementary Data S8).

#### Abbreviations of cancer types

A list of abbreviations of cancer types used in this study: ALL/CLL, acute/chronic lymphoblastic leukemia; BLCA, bladder urothelial carcinoma; BONE, bone cancer other/not classified further; COREAD, colon adenocarcinoma and rectum adenocarcinoma; COREAD_MSI, microsatellite instable colon and rectum adenocarcinoma; COREAD_MSS, microsatellite stable colon and rectum adenocarcinoma; ESCA, esophageal carcinoma; ESCA_STAD, esophagus carcinoma and stomach adenocarcinoma; EWING, Ewing's sarcoma; GBM, glioblastoma multiforme; GBM_LGG, glioblastoma and brain lower grade glioma; HNSC, head and neck squamous cell carcinoma; HNSC_LUSC, head and neck squamous cell carcinoma and lung squamous cell carcinoma; KIRC, kidney renal clear cell carcinoma; LAML, acute myeloid leukemia; LGG, brain lower grade glioma; LIHC, liver hepatocellular carcinoma; LUAD, lung adenocarcinoma; LUSC, lung squamous cell carcinoma; LYMP, lymphoma; MESO, mesothelioma; MM, multiple myeloma; NB, neuroblastoma; OV, ovarian serous cystadenocarcinoma; PAAD, pancreatic adenocarcinoma; SARC, sarcoma other/not classified further; SCLC, small cell lung cancer; SKCM, skin cutaneous melanoma; STAD, stomach adenocarcinoma; THCA, thyroid carcinoma; UCEC, uterine corpus endometrial carcinoma.

### Reporting summary

Further information on research design is available in the Nature Research Reporting Summary linked to this article.

## Supplementary information

Supplementary Information

Supplementary Tables

Reporting Summary

## Data Availability

The data sources used for this study are listed below and described further in the Methods. The data resulting from our analyses are available in the Supplementary Material, or are otherwise available from the authors upon request. Source data for figures are provided with this paper. Data sources used: WES bam files for human cancer cell lines (EGA ID number EGAD00001001039, restricted access that can be applied to following instructions on EGA), WES bam files for tumors and their matched normals (dbGaP accession ID phs000178 [https://www.ncbi.nlm.nih.gov/projects/gap/cgi-bin/study.cgi?study_id=phs000178.v11.p8], restricted access that can be applied to following instructions on dbGaP; bams downloaded from NCI Genomic Data Commons [https://portal.gdc.cancer.gov/]), drug response data for human cancer cell lines [https://www.cancerrxgene.org/]; Release 8.3), PRISM Repurposing dataset 19Q4 [https://depmap.org/portal/download/all/], Project Score CRISPR genetic screening data [https://score.depmap.sanger.ac.uk/downloads]; Integrated cancer dependency dataset from Wellcome Sanger Institute (release 1) and Broad Institute (19Q3)) [https://score.depmap.sanger.ac.uk/downloads]. Source data are provided with this paper.

## Source data

Source Data

## Code availability

The computer code is available at the GitHub repository: https://github.com/jlevatic/CellLineMutSigs (10.5281/zenodo.6463399)^109^.
