# Supplementary material for: Mutational signatures are markers of drug sensitivity of cancer cells
Source: Nat Commun. 2022 May 25;13:2926. doi: 10.1038/s41467-022-30582-3 (PMC9132939; doi:10.1038/s41467-022-30582-3)
Supplement: Supplementary file 1 — Supplementary Information [file 41467_2022_30582_MOESM1_ESM.pdf]

Levatić, Salvadores, Fuster-Tormo and Supek. **Mutational signatures are markers of drug sensitivity of cancer cells.** Supplementary Figures.

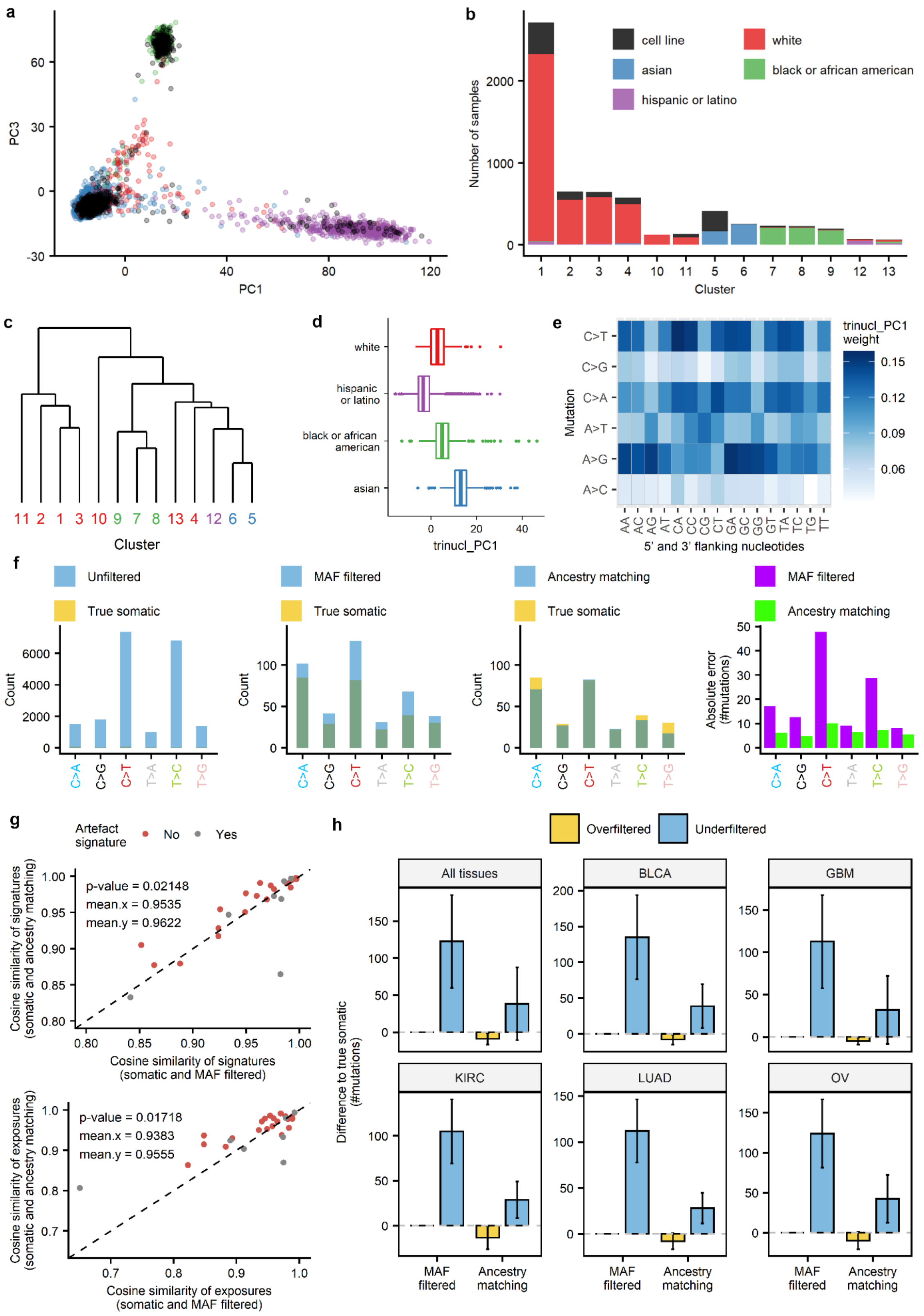

Supplementary Figure 1. Ancestry signal in cell line exomes is reflected in trinucleotide mutation spectra of rare germline variants, which can confound mutational signature analysis. a, PC analysis of common germline SNVs (population MAF>5%) of TCGA samples and cancer cell lines recovers the major ethnicity groups. **b**, Clustering based on the 140 main principal components (see Methods) reveals that cancer cell lines are represented across diverse genetic ancestry subgroups within the major ethnicity groups. **c**, Hierarchical clustering of the median trinucleotide spectra (derived from rare SNVs, MAF<0.001%, thus independent of the PC analysis) of the 13 ethnicity subgroups procedure shows that the intra-ethnicity tri-nucleotide profiles bear more similarity to each other than intra-ethnicity profiles (the subgroup labels are colored by the prevalent ethnicity within the cluster, with colors corresponding to panel **b**). **d**, **e**, The main component of the PC analysis of tri-nucleotide spectra of the very rare germline variants (i.e., those that often cannot be removed by filtering using population databases; here MAF<0.001% or absent in gnomAD) separates the major ethnicity groups (n=4004 cancer exomes from white ethnicity, 149 from hispanic or latino, 604 from black or african american, and 414 from asian). The center line of box plots denotes medians of all data points and the box hinges correspond to the 1st and 3rd quartiles, while whiskers extend to 1.5 × IQR from the hinges. Data points beyond the end of the whiskers are shown individually. **f**, Average 6 mutation types spectra of 450 simulated cell lines. True somatic mutations are overlaid with the MAF unfiltered spectra, spectra obtained by the commonly used approach of filtering (MAF<0.001%) according to the population databases (2nd panel) and the spectra obtained with the ‘ancestry matching’ method (3rd panel). Average error of reconstruction of the spectra of 6 mutation types with MAF filtering and the ‘ancestry matching’ method (4th panel). **g**, Cosine similarity between mutational signatures and their exposures extracted from 450 simulated cell lines using true somatic mutations, the mixture of somatic mutations and germline variants and the common approach for removal of germline variants (MAF filtering), and the ‘ancestry matching’ approach. The signatures obtained with the ‘ancestry matching’ approach more closely resemble (by both 96 tri-nucleotide profiles and exposure attributions) the signatures obtained with the true somatic mutations suggesting better filtering of germline variants. P-values are from the two-sided Wilcoxon rank sum test. **h**, The comparison of the magnitudes (mean ± SD) of overfiltration vs. underfiltration of MAF filtering and ‘ancestry matching’ approaches shows the overfiltration of the ‘ancestry matching’ is much smaller in magnitude than the underfiltering of the MAF filtering approach, while also exhibiting ~2x smaller underfiltering (n=450 cancer exomes). Source data are provided as a Source Data file.

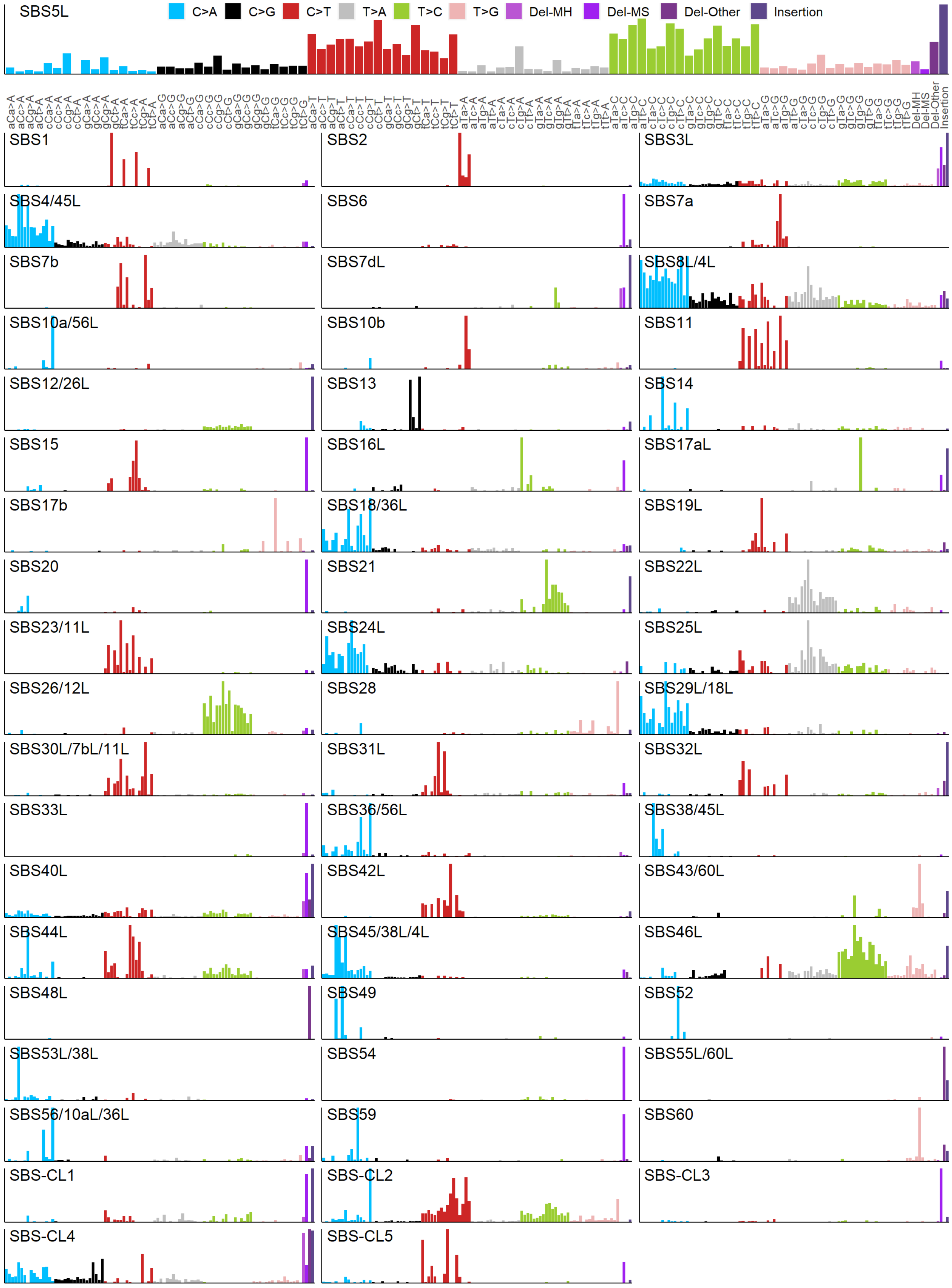
SBS5L
C>A
C>G
C>T
T>A
T>C
T>G
Del-MH
Del-MS
Del-Other
Insertion
SBS1
SBS2
SBS3L
SBS4/45L
SBS6
SBS7a
SBS7b
SBS7dL
SBS8L/4L
SBS10a/56L
SBS10b
SBS11
SBS12/26L
SBS13
SBS14
SBS15
SBS16L
SBS17aL
SBS17b
SBS18/36L
SBS19L
SBS20
SBS21
SBS22L
SBS23/11L
SBS24L
SBS25L
SBS26/12L
SBS28
SBS29L/18L
SBS30L/7bL/11L
SBS31L
SBS32L
SBS33L
SBS36/56L
SBS38/45L
SBS40L
SBS42L
SBS43/60L
SBS44L
SBS45/38L/4L
SBS46L
SBS48L
SBS49
SBS52
SBS53L/38L
SBS54
SBS55L/60L
SBS56/10aL/36L
SBS59
SBS60
SBS-CL1
SBS-CL2
SBS-CL3
SBS-CL4
SBS-CL5

39

Supplementary Figure 2. The 96 trinucleotide and 4 indel mutational spectra of the cell line signatures inferred in this study. “Del-MH” are deletions with microhomology, “Del-MS” are deletions at microsatellite loci, “Del-Other” are other deletions, while “Insertion” are insertions at any locus. The y-axis corresponds to NMF weights. Source data are provided as a Source Data file.

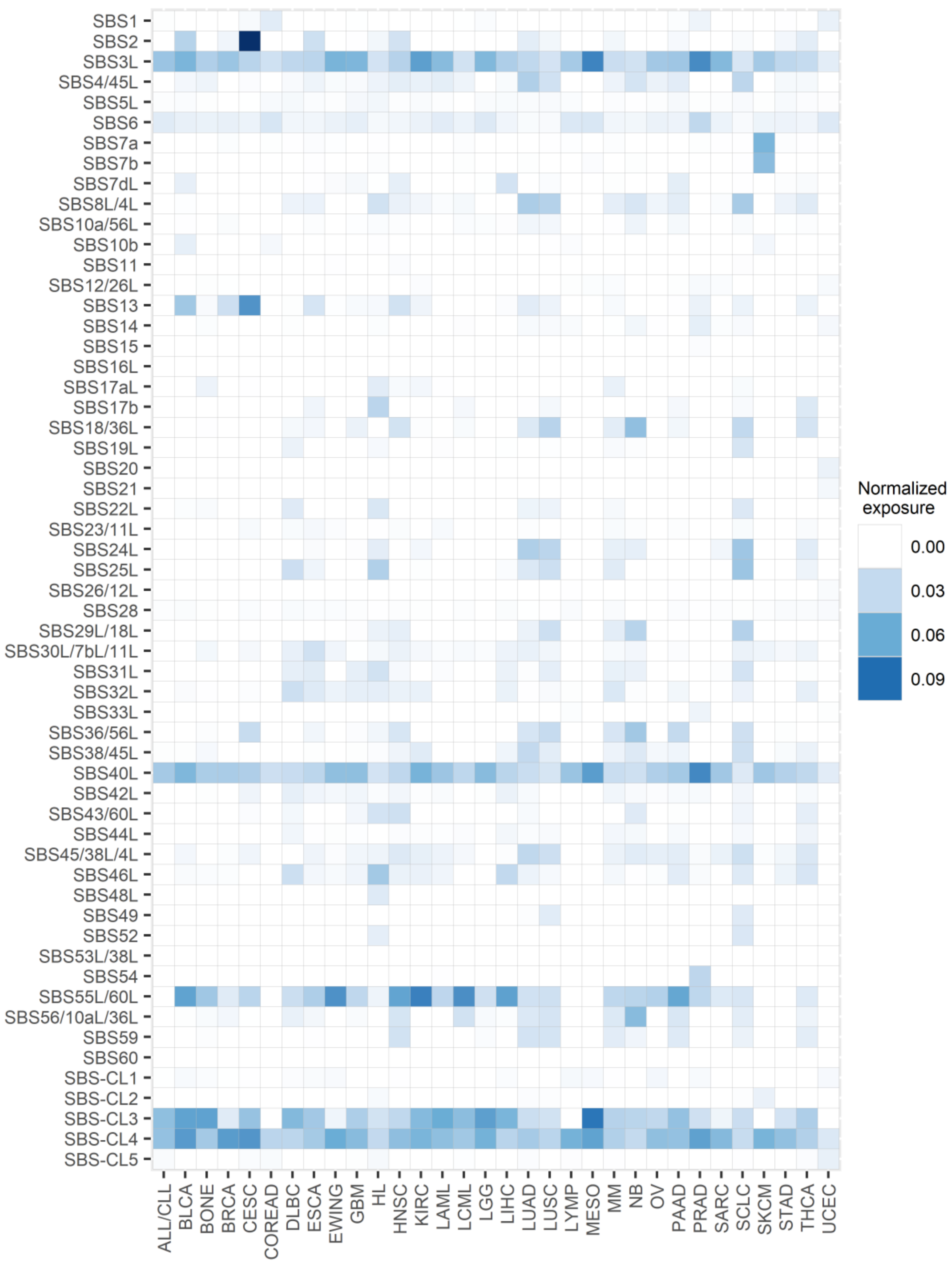

Supplementary Figure 3. Estimated exposures of tissues-of-origin of the cancer cell lines to mutational signatures inferred in this work. The color intensity in the heatmap corresponds to the median NMF score per cancer type (only cancer types represented by at least 5 cell lines are shown). For each cell line, the scores were normalized to represent the

relative contribution of each signature in the exome of that cell line. Source data are provided as a Source Data file.

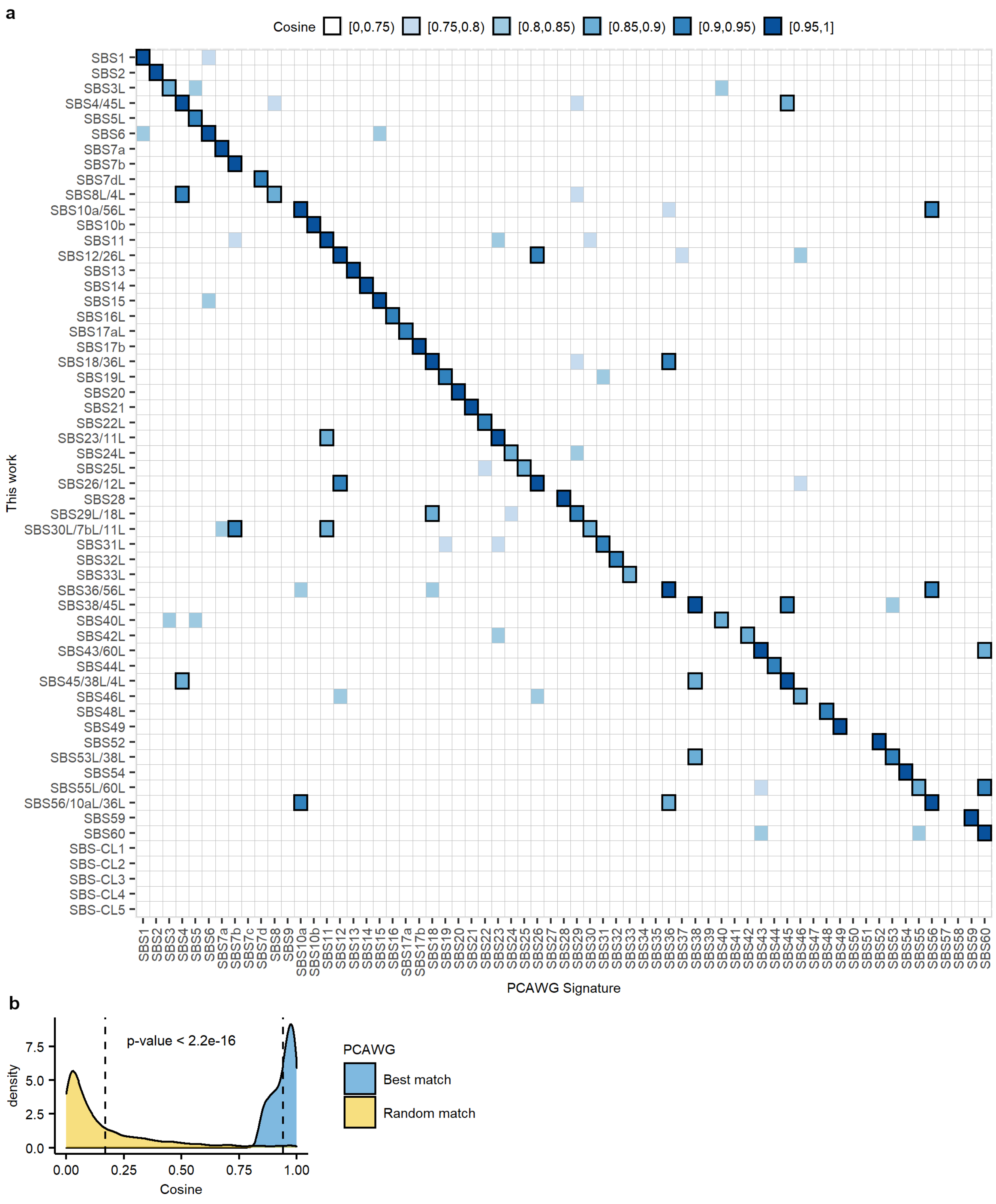

Supplementary Figure 4. Cell line mutational signatures sometimes map to multiple tumor mutational signatures. a, Cosine similarity between cell line mutational signatures and known tumor mutational signatures. Some of the cell line signatures discovered in this work are highly similar (cosine>0.85) to more than one tumor signature (marked with bold black border rectangles). Such ambiguity is made clear in the naming convention of the cell line signatures where all of the highly resembling tumor signatures are included in the name of the cell line signature. Signatures that closely match previous tumor signatures (cosine similarity >= 0.95) are denoted with a corresponding name of the SBS tumor signature, the suffix “L” (for like) denotes a less close match (0.85 <= cosine similarity < 0.95). Cancer cell line specific signatures (denoted as SBS-CL) are dissimilar to any of the tumor signatures and may represent cell-line specific mutational processes, germline mutations, or artefacts. **b**, The density plot of cosine similarities of best matches of each of our cell line signatures to the PCAWG signatures, contrasted against a randomized baseline where each of our signatures is randomly matched to a PCAWG signature (repeated 100 times). The p-value of Mann-Whitney test comparing the means of the two distributions is shown. Source data are provided as a Source Data file.

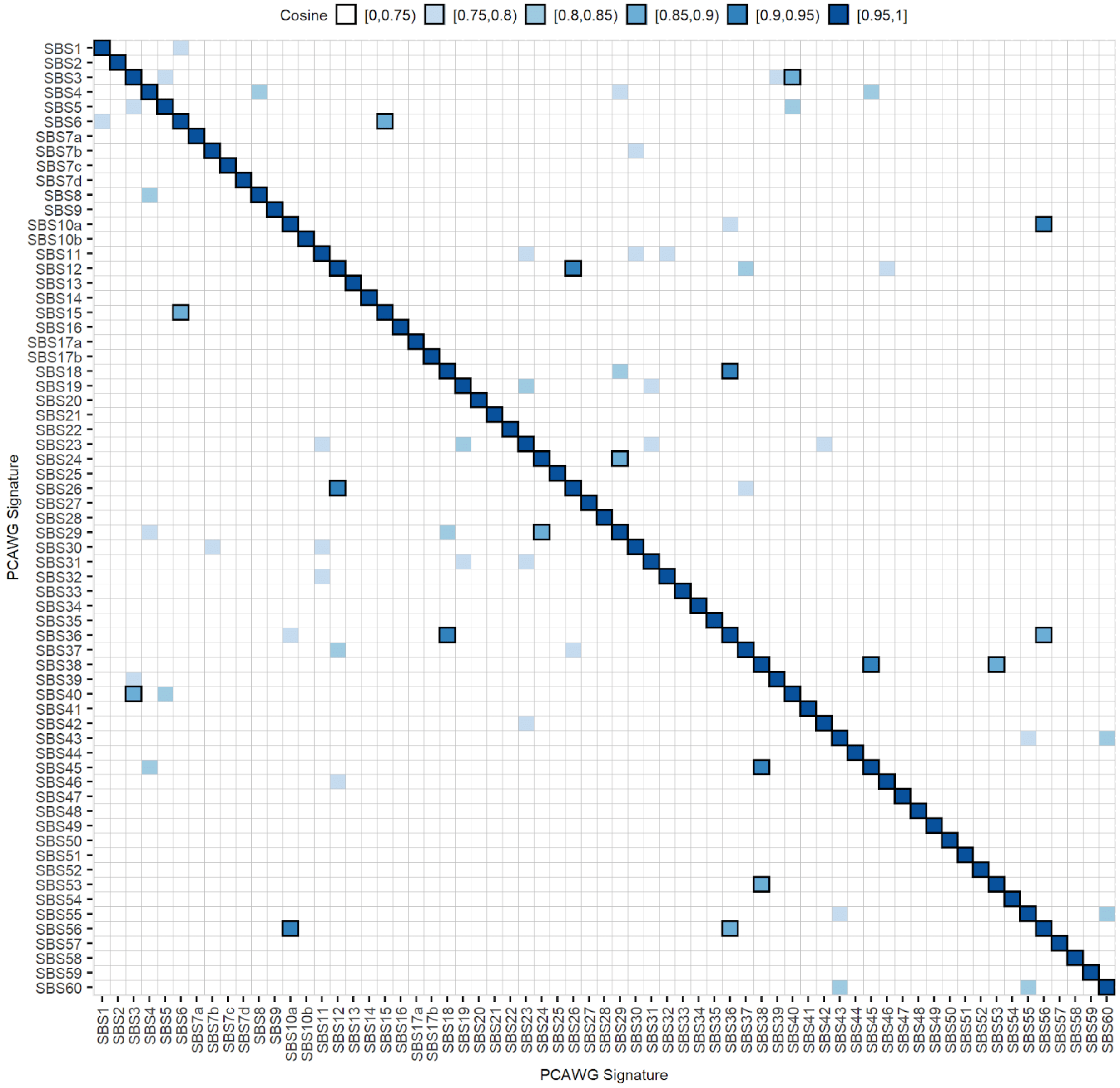

Supplementary Figure 5. **The known tumor mutational signatures may have similar mutation spectra to other known tumor mutational signatures.** Cosine similarity between known tumor mutational signatures shows that some of the reported signatures are highly similar to each other, such as SBS6 and 15 (cosine=0.86), or SBS12 and 26 (cosine=0.93). Cosine similarities greater than 0.85 are marked with black borders. Source data are provided as a Source Data file.

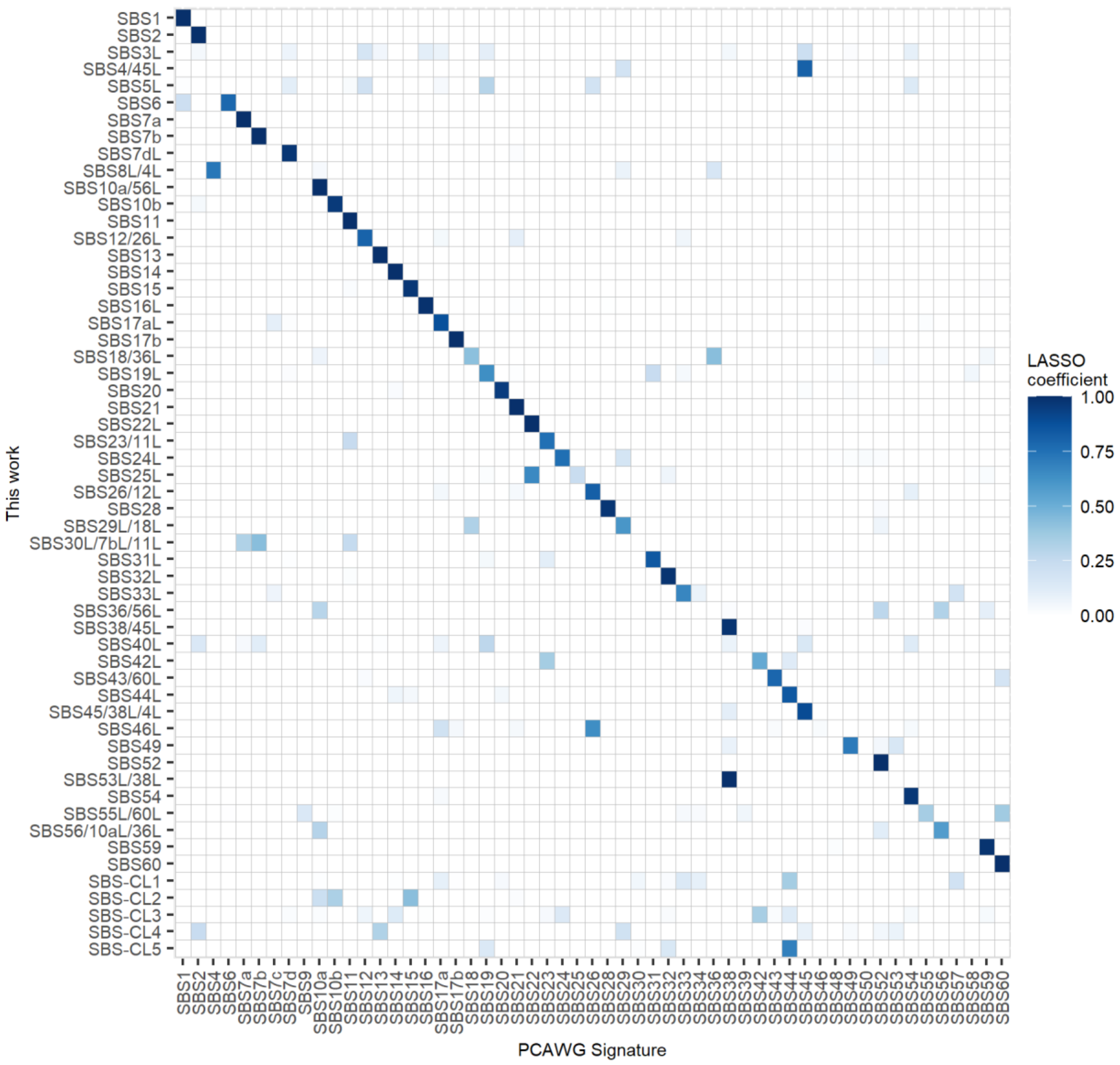

Supplementary Figure 6. Modelling cell line mutation signatures as mixtures of tumor mutation signatures. Coefficients of LASSO linear regression where cell line mutational signatures are represented as a linear combination of known tumor mutational signatures reported by the PCAWG consortium. For example, the 96-nucleotide spectrum of our cell line SBS-CL4 (penultimate row) may be modelled as a mixture of tumor SBSs (columns) 2, 13 and minor contributions of other signatures (primarily 29 and 45), while SBS-CL5 may be modelled as a mixture of SBS19, 32 and 44. Source data are provided as a Source Data file.

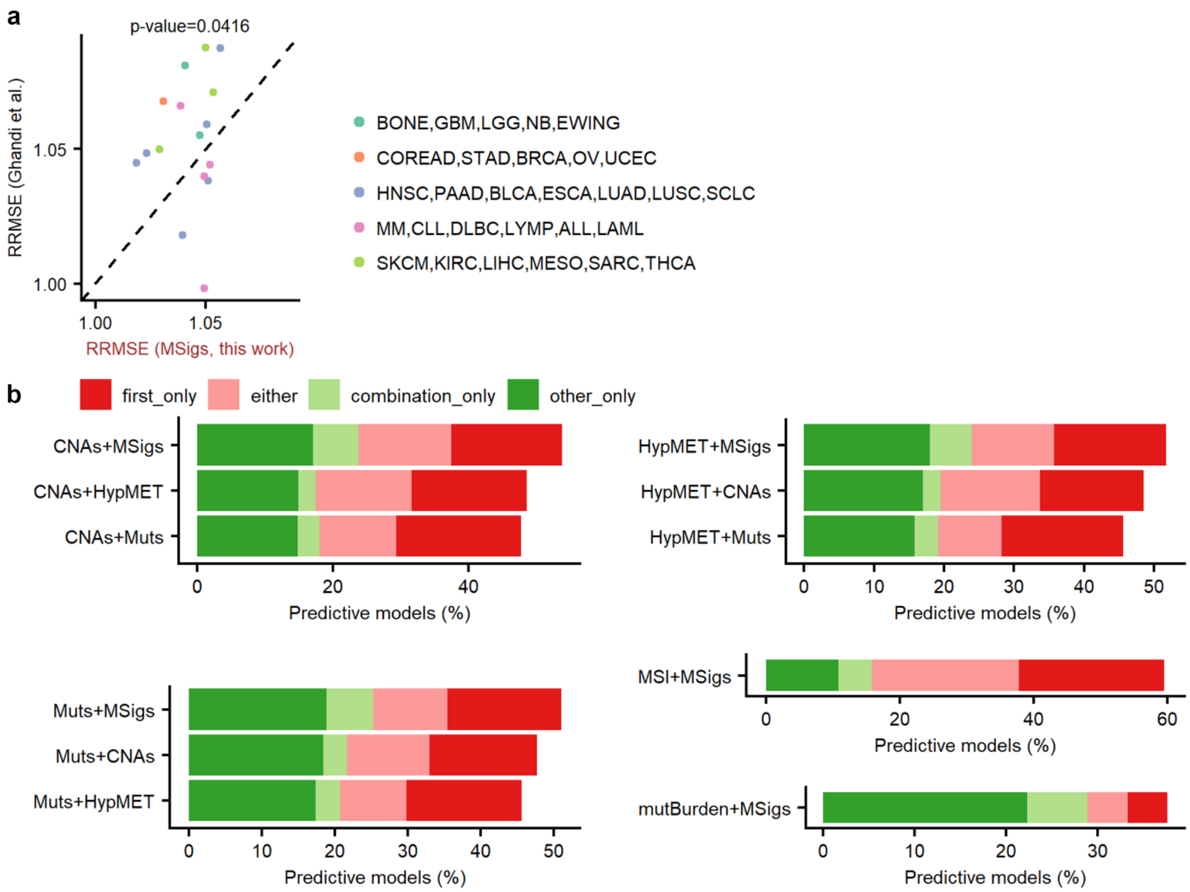

Supplementary Figure 7. Complementarity of drug response prediction with mutational signatures and other molecular data types. a, Predictive performance (RRMSE) of drug response prediction using Random Forest models based on mutational signatures (MSigs) reported here and previously. The 667 overlapping cell lines from 16 cancer types between this study and the study of Ghandi *et al.* were considered. P-value of paired Wilcoxon signed rank test (one-sided) is reported. The dashed line denotes the diagonal. **b**, Complementarity between mutational signatures reported here and other data types (copy number alterations, oncogenic mutations, DNA hypermethylation, the MSI/MSS status of cell lines, and a total mutational burden of cell lines. For each combination of data types, bars show the percentages of Random Forest models that are predictive (below-baseline RMSE) with mutational signatures but not another data type ("other_only"), by other feature type but not with mutational signatures ("first_only"), individually by either data type ("either"), or only by a combination of both data types ("combination_only"). The MSI+MSigs bar shows results only for 5 cancer types (colorectal, ovarian, stomach, uterine, and acute/chronic lymphoblastic leukemia) where MSI/MSS labels were available, while other panels show results for all cancer types. Source data are provided as a Source Data file.

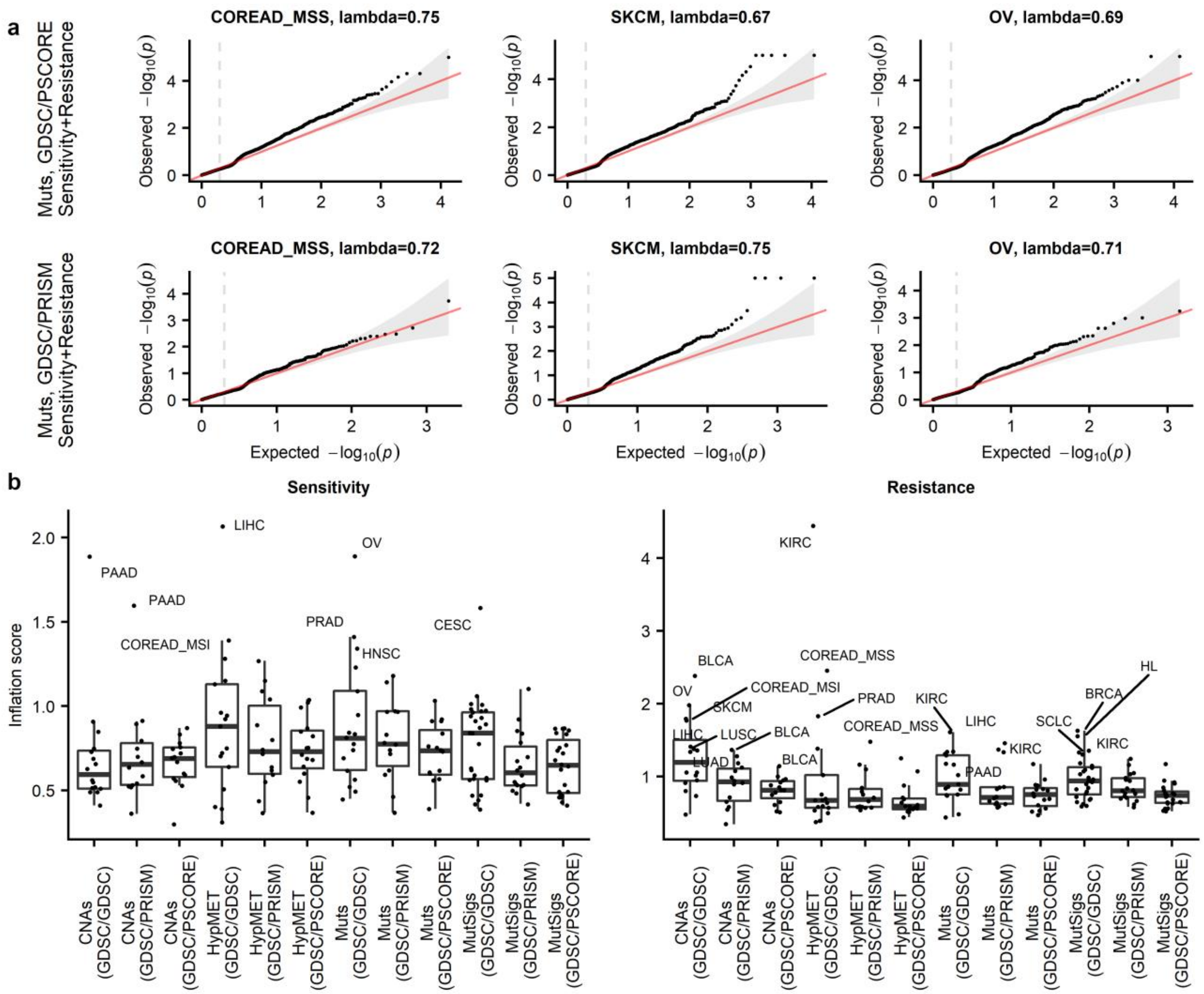

Supplementary Figure 8. Calibration of p-values in the randomization tests for replication of drug-marker associations. a, Examples of q-q plots for selected cancer types for GDSC/PSCORE and GDSC/PRISM “two-way” replication tests (see Methods). The inflation factor (lambda) is denoted for each q-q plot.The vertical dashed line denotes the median. The error bands denote the 95% confidence interval. **b**, Inflation factors across all cancer types, two-way tests and feature types (Muts, CNAs, HypMET, and mutational signatures) generally exhibit a deflation of p-values (lambda<1), while a few cancer types have inflated p-values (lambda>1.3; cancer types denoted on the plot) in some two-way tests and with some feature types (n=29 cancer types). The center line of box plots denotes medians of all data points and the box hinges correspond to the 1st and 3rd quartiles, while whiskers extend to 1.5 × IQR from the hinges. Source data are provided as a Source Data file.

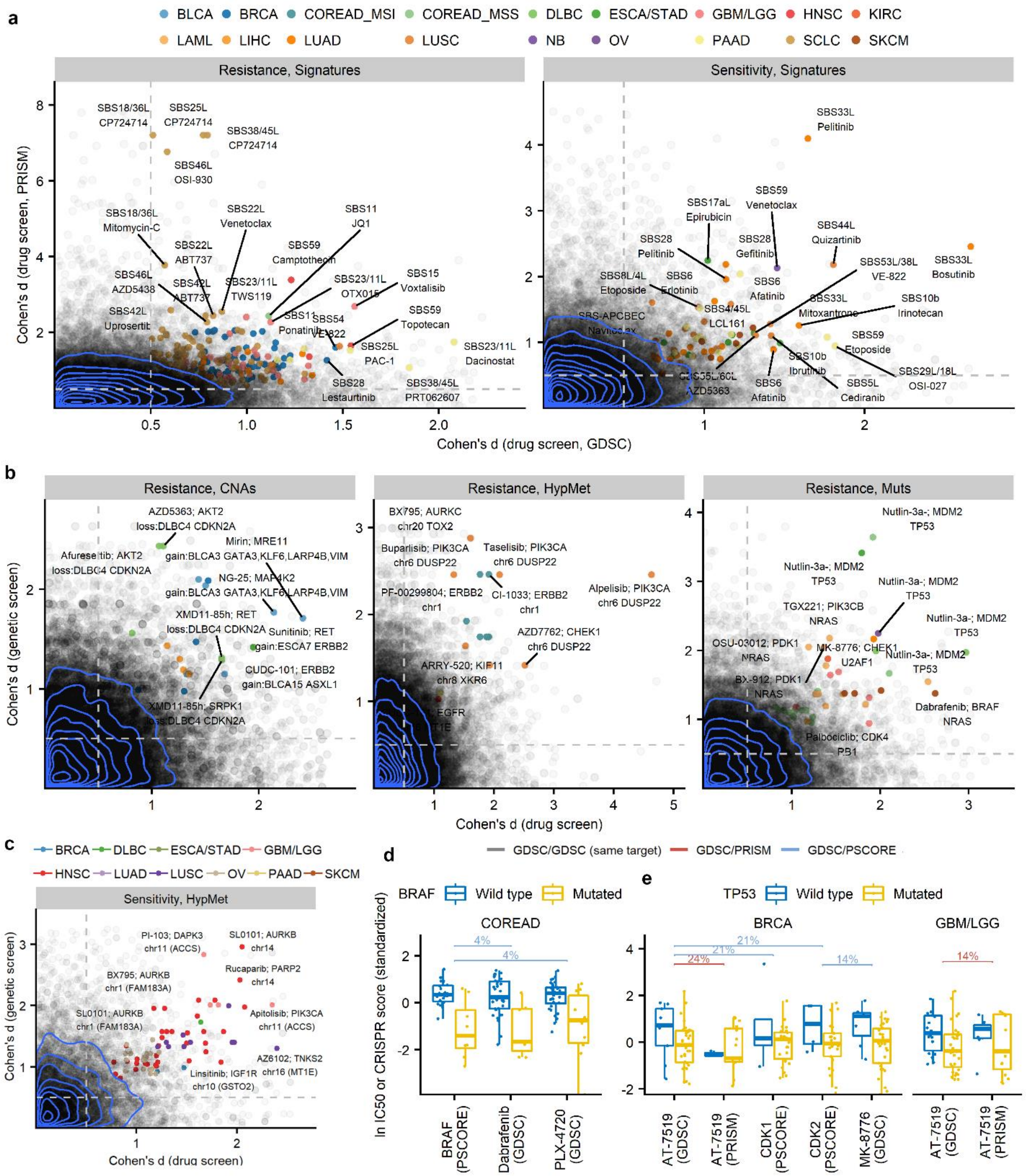

Supplementary Figure 9. Associations that replicate in independent datasets. a, The GDSC/PRISM replication test, which requires that the association between mutational signature and drug sensitivity or resistance replicates in the PRISM data set based on pooled cell line screening for drug response. Gray points represent all tested associations, while colored points denote the statistically significant associations according to a randomization test (Methods) and that also meet the required effect size threshold of Cohen's *d*>0.5. Blue lines are the contours of the 2D kernel density estimates. **b**, **c**, Plots as in **a**, but for replication of drug resistance (**b**) and

drug sensitivity (**c**) associations (X-axis) in genetic screening data (Project SCORE, Y-axis) for classical markers: oncogenic mutations (Muts), copy number alterations (CNAs) and DNA hypermethylation (HypMet). **d**, **e**, Examples of replicated associations of a known example of oncogene addiction (to *BRAF*, **b**) and of novel examples of cancer vulnerabilities associated with mutations in tumor suppressor genes (*TP53*, **d**). Y-axes show a Z-score derived from either the ln IC50 value (drug sensitivity) or from the CRISPR essentiality score, depending on column. Horizontal bars show false discovery rates (q-value adjusted) obtained via a randomization test in panel **a**, where color denotes the type of the test. The center line of box plots denotes medians of all data points and the box hinges correspond to the 1st and 3rd quartiles, while whiskers extend to 1.5 × IQR from the hinges. Source data are provided as a Source Data file.

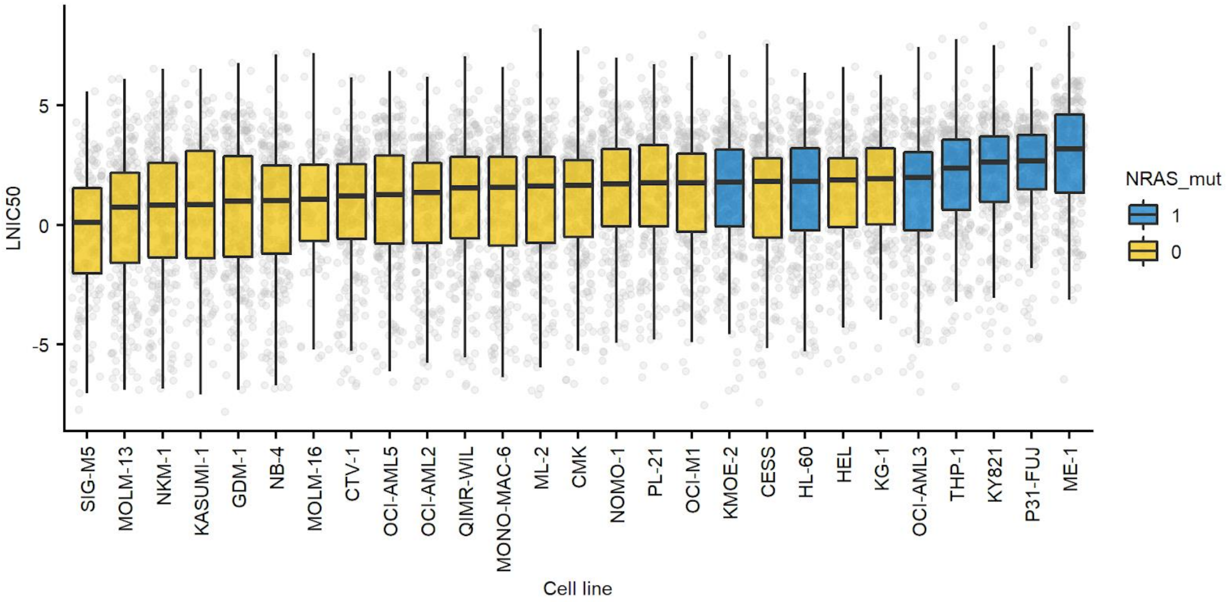

Supplementary Figure 10. **Resistance of acute myeloid leukemia cell lines to diverse drugs**. Cell lines are sorted by median log IC50 calculated across all drugs screened against a cell line. NRAS mutant cell lines show (blue) an median resistance to many diverse drugs compared to NRAS wild-type cells, suggesting a multidrug resistance phenotype. The center line of box plots denotes medians of all data points and the box hinges correspond to the 1st and 3rd quartiles, while whiskers extend to 1.5 × IQR from the hinges. Source data are provided as a Source Data file.

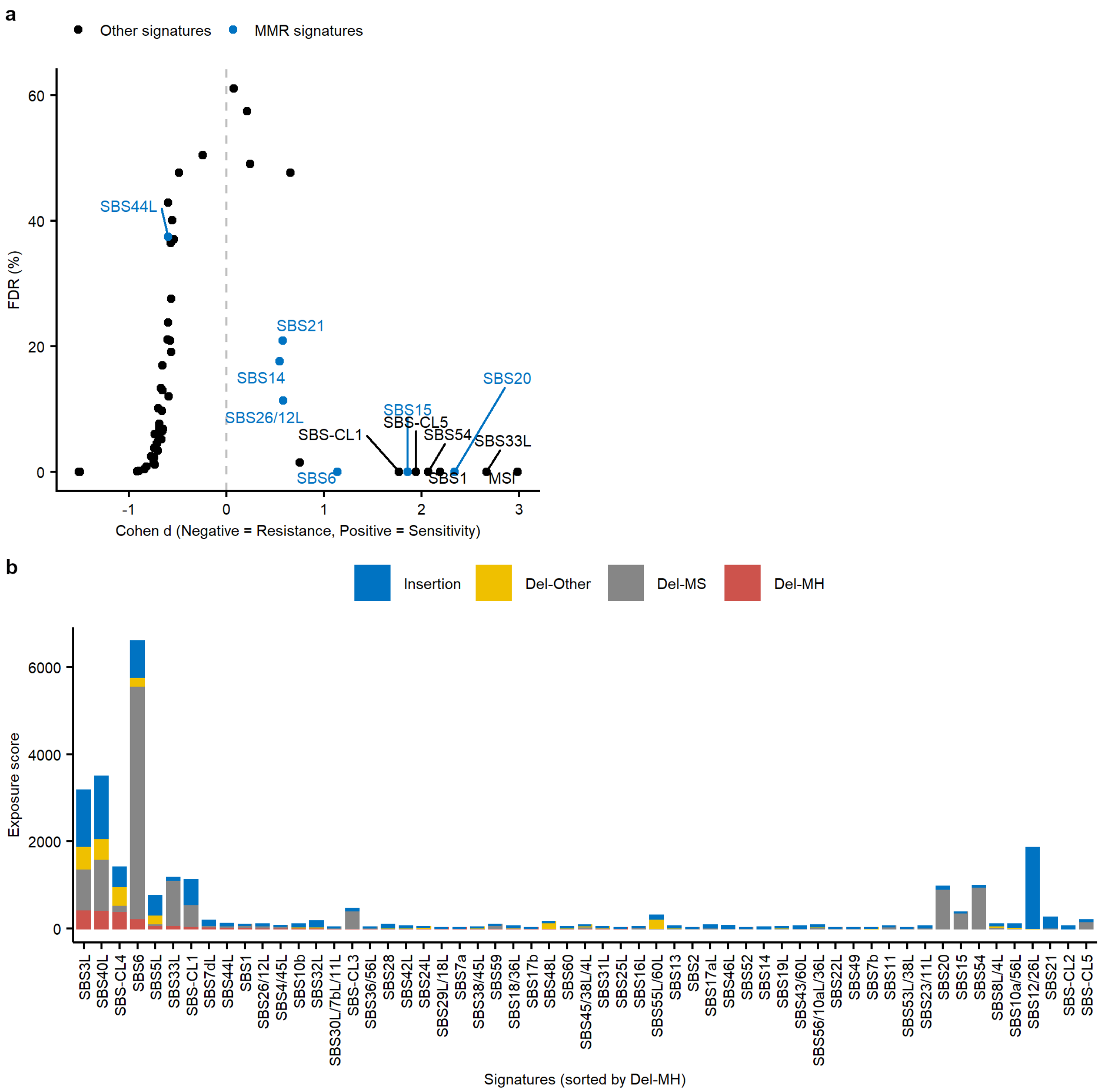

Supplementary Figure 11. Additional analyses related with MMR status. a, Different MMR-failure associated mutational signatures predict sensitivity to knock-out of the WRN gene to different extents. FDR of associations between different mutational signatures (including the MSI status of cell lines) and knock-out of the WRN gene in a pan-cancer analysis. The effect size is Cohen's d statistic. **b**, The exposure scores of the indel components of cell line mutational signatures. Source data are provided as a Source Data file.

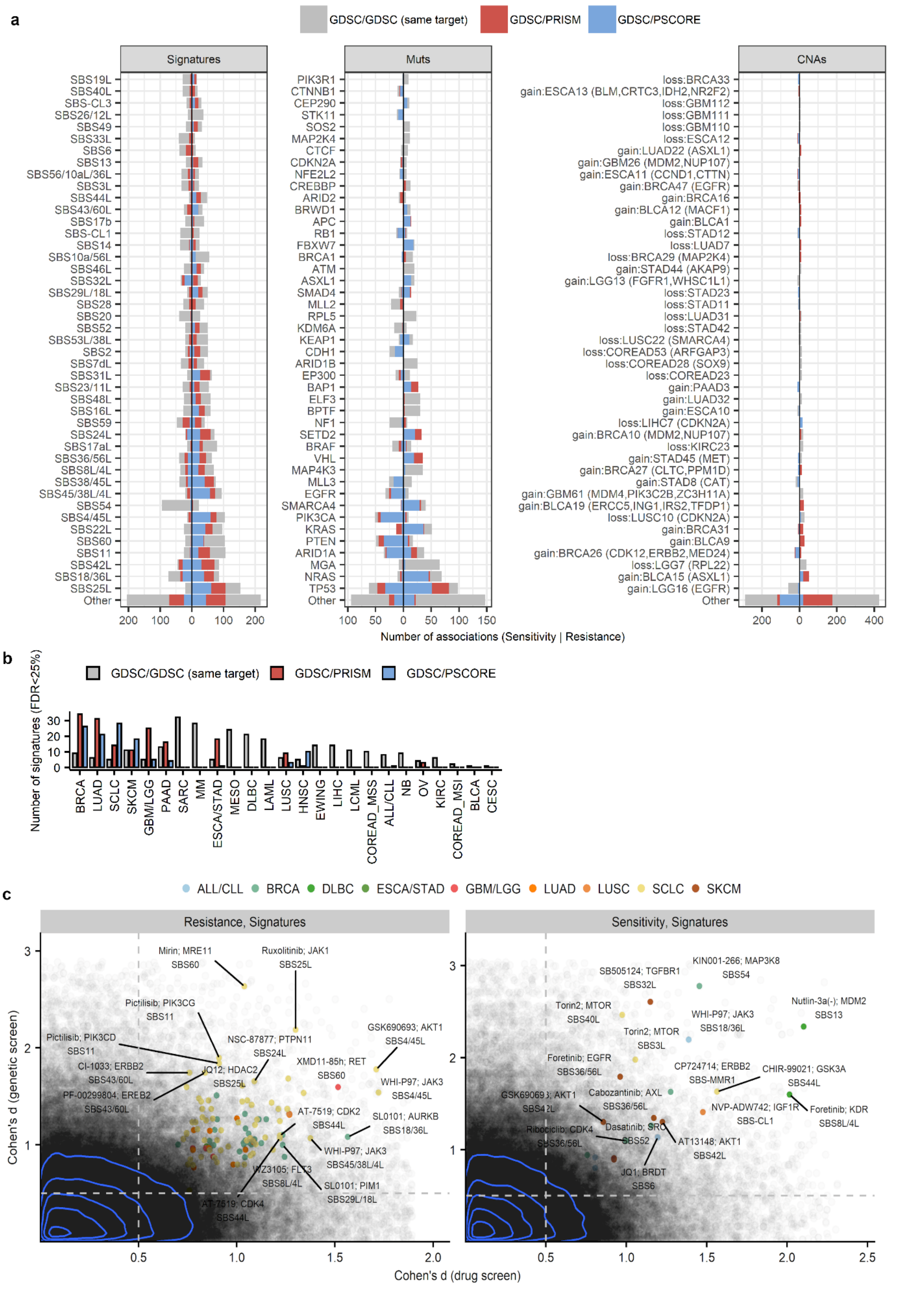
a
GDSC/GDSC (same target)
GDSC/PRISM
GDSC/PSCORE
Signatures
SBS19L
SBS40L
SBS-CL3
SBS26/12L
SBS49
SBS33L
SBS6
SBS13
SBS56/10aL/36L
SBS3L
SBS44L
SBS43/60L
SBS17b
SBS-CL1
SBS14
SBS10a/56L
SBS46L
SBS32L
SBS29L/18L
SBS28
SBS20
SBS52
SBS53L/38L
SBS2
SBS7dL
SBS31L
SBS23/11L
SBS48L
SBS16L
SBS59
SBS24L
SBS17aL
SBS36/56L
SBS8L/4L
SBS38/45L
SBS45/38L/4L
SBS54
SBS4/45L
SBS22L
SBS60
SBS11
SBS42L
SBS18/36L
SBS25L
Other
200 100 0 100 200
Muts
PIK3R1
CTNNB1
CEP290
STK11
SOS2
MAP2K4
CTCF
CDKN2A
NFE2L2
CREBBP
ARID2
BRWD1
APC
RB1
FBXW7
BRCA1
ATM
ASXL1
SMAD4
MLL2
RPL5
KDM6A
KEAP1
CDH1
ARID1B
EP300
BAP1
ELF3
BPTF
NF1
SETD2
BRAF
VHL
MAP4K3
MLL3
EGFR
SMARCA4
PIK3CA
KRAS
PTEN
ARID1A
MGA
NRAS
TP53
Other
100 50 0 50 100 150
CNAs
loss:BRCA33
gain:ESCA13 (BLM,CRTC3,IDH2,NR2F2)
loss:GBM112
loss:GBM111
loss:GBM110
loss:ESCA12
gain:LUAD22 (ASXL1)
gain:GBM26 (MDM2,NUP107)
gain:ESCA11 (CCND1,CTTN)
gain:BRCA47 (EGFR)
gain:BRCA16
gain:BLCA12 (MACF1)
gain:BLCA1
loss:STAD12
loss:LUAD7
loss:BRCA29 (MAP2K4)
gain:STAD44 (AKAP9)
gain:LGG13 (FGFR1,WHSC1L1)
loss:STAD23
loss:STAD11
loss:LUAD31
loss:STAD42
loss:LUSC22 (SMARCA4)
loss:COREAD53 (ARFGAP3)
loss:COREAD28 (SOX9)
loss:COREAD23
gain:PAAD3
gain:LUAD32
gain:ESCA10
loss:LIHC7 (CDKN2A)
gain:BRCA10 (MDM2,NUP107)
loss:KIRC23
gain:STAD45 (MET)
gain:BRCA27 (CLTC,PPM1D)
gain:STAD8 (CAT)
gain:GBM61 (MDM4,PIK3C2B,ZC3H11A)
gain:BLCA19 (ERCC5,ING1,IRS2,TFDP1)
loss:LUSC10 (CDKN2A)
gain:BRCA31
gain:BLCA9
gain:BRCA26 (CDK12,ERBB2,MED24)
loss:LGG7 (RPL22)
gain:BLCA15 (ASXL1)
gain:LGG16 (EGFR)
Other
200 0 200 400
Number of associations (Sensitivity | Resistance)
b
GDSC/GDSC (same target)
GDSC/PRISM
GDSC/PSCORE
Number of signatures (FDR<25%)
30
20
10
0
BRCA
LUAD
SCLC
SKCM
GBM/LGG
PAAD
SARC
MM
ESCA/STAD
MESO
DLBC
LAML
LUSC
HNSC
EWING
LIHC
LCML
COREAD_MSS
ALL/CLL
NB
OV
KIRC
COREAD_MSI
BLCA
CESC
c
ALL/CLL
BRCA
DLBC
ESCA/STAD
GBM/LGG
LUAD
LUSC
SCLC
SKCM
Resistance, Signatures
Mirin; MRE11
SBS60
Ruxolitinib; JAK1
SBS25L
Pictilisib; PIK3CG
SBS11
Pictilisib; PIK3CD
SBS11
NSC-87877; PTPN11
SBS24L
GSK690693; AKT1
SBS4/45L
CI-1033; ERBB2
SBS43/60L
JQ12; HDAC2
SBS25L
XMD11-85h; RET
SBS60
PF-00299804; ERBB2
SBS43/60L
WHI-P97; JAK3
SBS4/45L
AT-7519; CDK2
SBS44L
SL0101; AURKB
SBS18/36L
WHI-P97; JAK3
SBS45/38L/4L
WZ3105; FLT3
SBS8L/4L
SL0101; PIM1
SBS29L/18L
AT-7519; CDK4
SBS44L
Sensitivity, Signatures
SB505124; TGFBR1
SBS32L
KIN001-266; MAP3K8
SBS54
Nutlin-3a(-); MDM2
SBS13
Torin2; MTOR
SBS40L
WHI-P97; JAK3
SBS18/36L
Torin2; MTOR
SBS3L
Foretinib; EGFR
SBS36/56L
CP724714; ERBB2
SBS-MMR1
CHIR-99021; GSK3A
SBS44L
GSK690693; AKT1
SBS42L
Cabozantinib; AXL
SBS36/56L
NVP-ADW742; IGF1R
SBS-CL1
Foretinib; KDR
SBS8L/4L
Ribociclib; CDK4
SBS36/56L
Dasatinib; SRC
SBS52
AT13148; AKT1
SBS42L
JQ1; BRDT
SBS6
Cohen's d (genetic screen)
Cohen's d (drug screen)
3
2
1
0.5 1.0 1.5 2.0 2.5

169

Supplementary Figure 12. Tally of the significantly replicated associations of drug sensitivity or resistance with mutation signatures and other markers. a, Comparison of the number of statistically significant associations (FDR<25% by randomization test, additionally requiring an effect size *d*>0.5 for GDSC/PRISM and GDSC/PSCORE tests, and d>1 for the GDSC/GDSC (same target) test) per feature, among mutational signatures, oncogenic mutations and copy number alterations. Features are ranked by the number total of significant associations, either for drug sensitivity (negative side of X-axis) or resistance (positive side of X-axis). **b**, The number of different mutational signatures that have statistically significant associations across different cancer types (at FDR<25%; we consider signatures that have >1 significant association per cancer type) in the three replication tests. **c**, The GDSC/PSCORE replication test of mutational signatures that robustly associate with drug response (X-axes) and also with response to the knock-out of the drug target gene (Y-axes). Gray points represent all tested associations, while colored points denote the statistically significant associations that also meet the effect size threshold of *d*>0.5. Blue lines are the contours of the 2D kernel density estimates. Representative points are labeled with drug name, gene name and SBS mutational signature involved in the association. Source data are provided as a Source Data file.

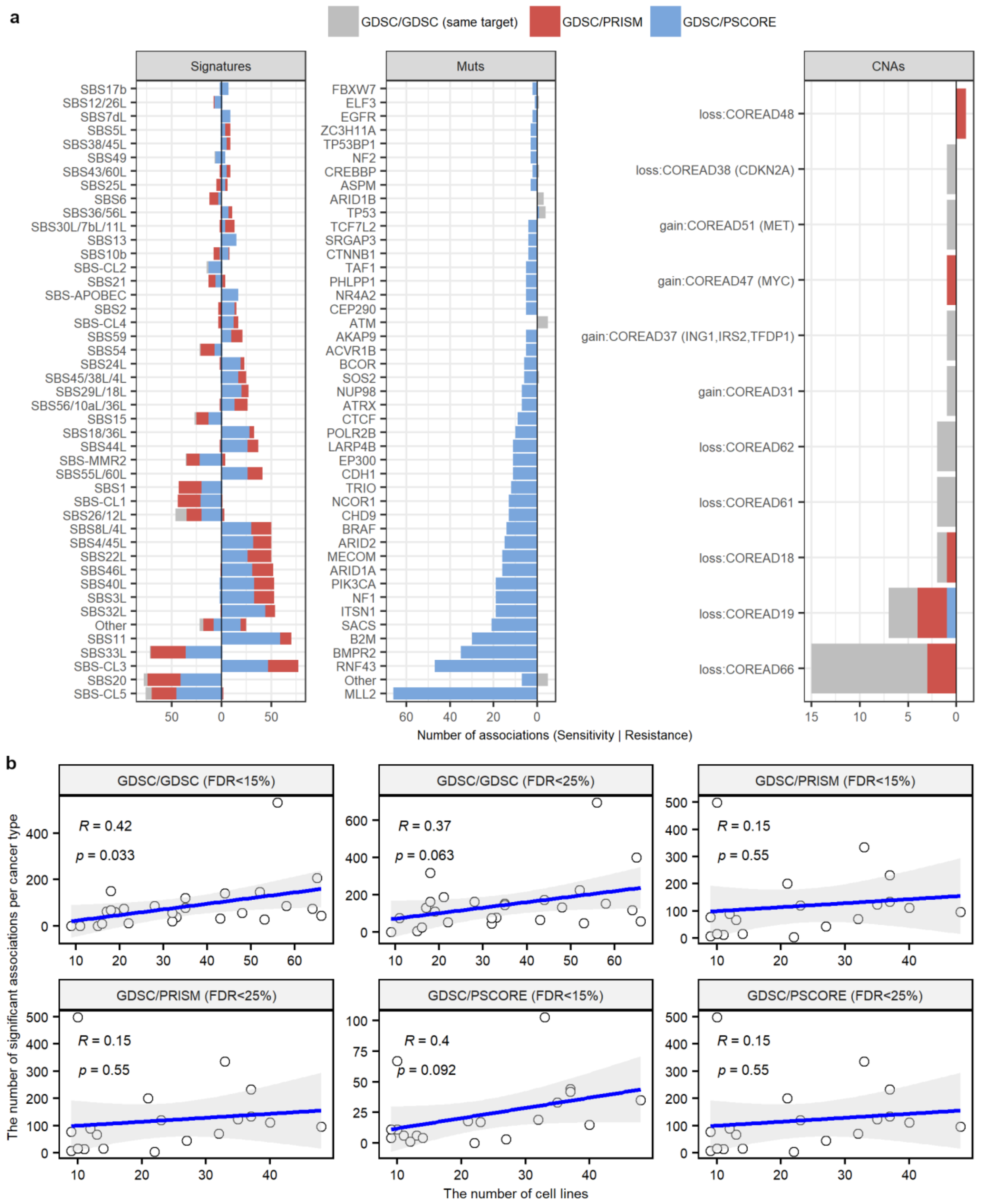

Supplementary Figure 13. Additional analyses of marker-drug associations that replicate across independent data sets. a, Tally of the significantly replicated associations (via the "two-way" randomization test, see Methods) with mutation signatures and other markers for colorectal cell lines, here without stratifying by MSI status. Comparison of the number of statistically significant associations (FDR<25%, effect size d>0.5 for GDSC/PRISM and

GDSC/PSCORE tests, and d>1 for GDSC/GDSC (same target) test) per feature, among mutational signatures, oncogenic mutations and copy number alterations in the three replication tests for colorectal cell lines (COREAD). Features are ranked by the number total of significant associations, either for drug sensitivity (negative side of X-axis) or resistance (positive side of X-axis). **b**, The number of statistically significant associations and the number of cell lines in the available different cancer types show positive Pearson correlation (R; p denotes p-value of two-sided t-test) across the three different types of ”two-way” randomization tests for replication (Methods). Associations in tissues that exhibit inflation of p-values (lambda>1.3; see Supplementary Fig. 8) were excluded. The error bands denote 95% confidence interval. Source data are provided as a Source Data file.

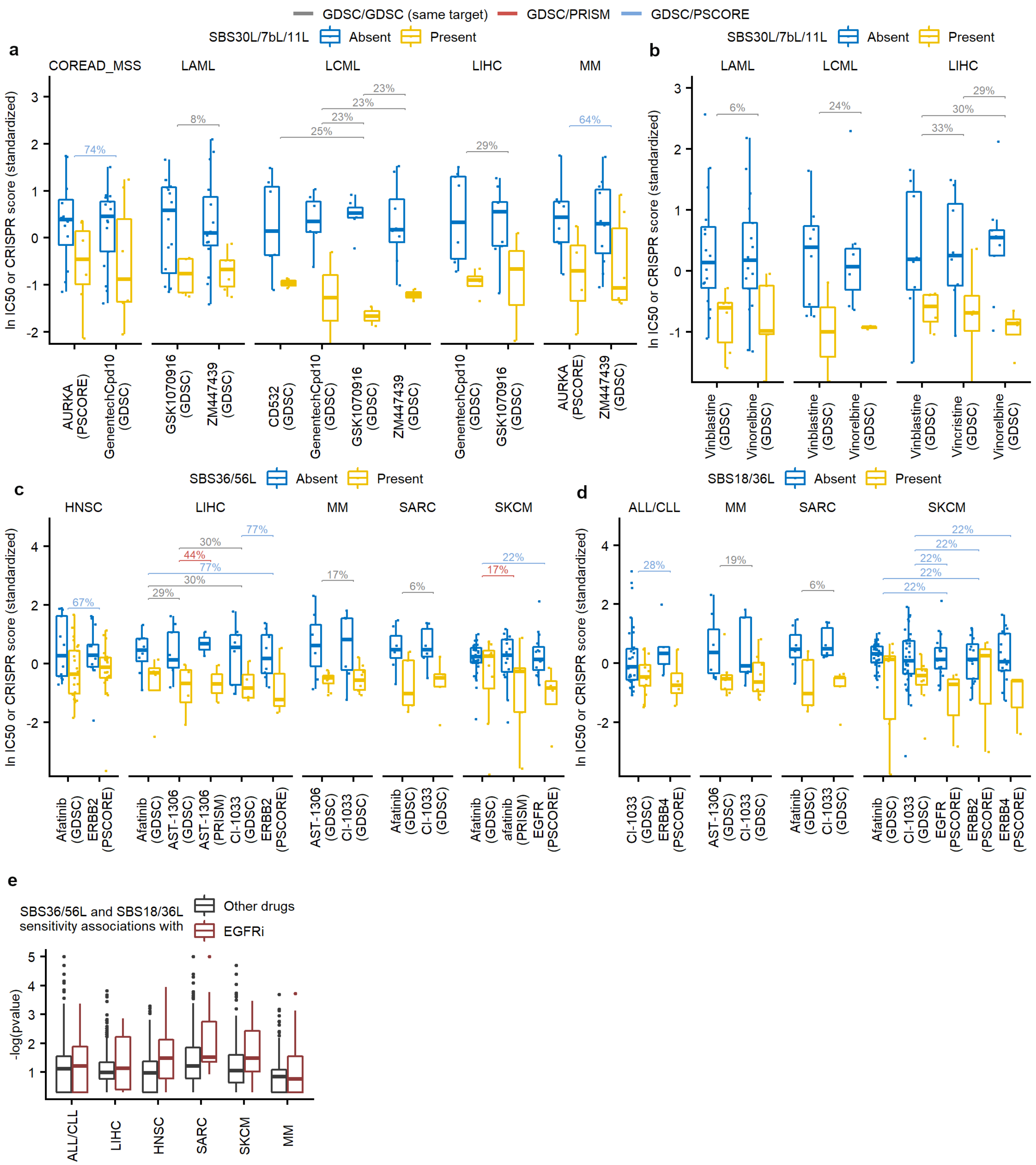

Supplementary Figure 14. Additional examples of significant associations involving mutational signatures that replicate in independent datasets. a, Association of SBS30L/7bL/11L with sensitivity to inhibitors of Aurora kinases a and b, and **b** with sensitivity to microtubule destabilising agents. FDRs via the “two-way” randomization test for replication across data sets (Methods). **c**, Association of SBS36/56L with sensitivity to sensitivity to FLT inhibitors and **d** inhibitors of ERBB2. **e**, The distribution of p-values of all tested sensitivity associations across the three replication tests involving the two mutational signatures related to

reactive oxygen species (SBS36/56L and SBS18/36L). The center line of box plots denotes medians of all data points and the box hinges correspond to the 1st and 3rd quartiles, while whiskers extend to 1.5 × IQR from the hinges. Source data are provided as a Source Data file.

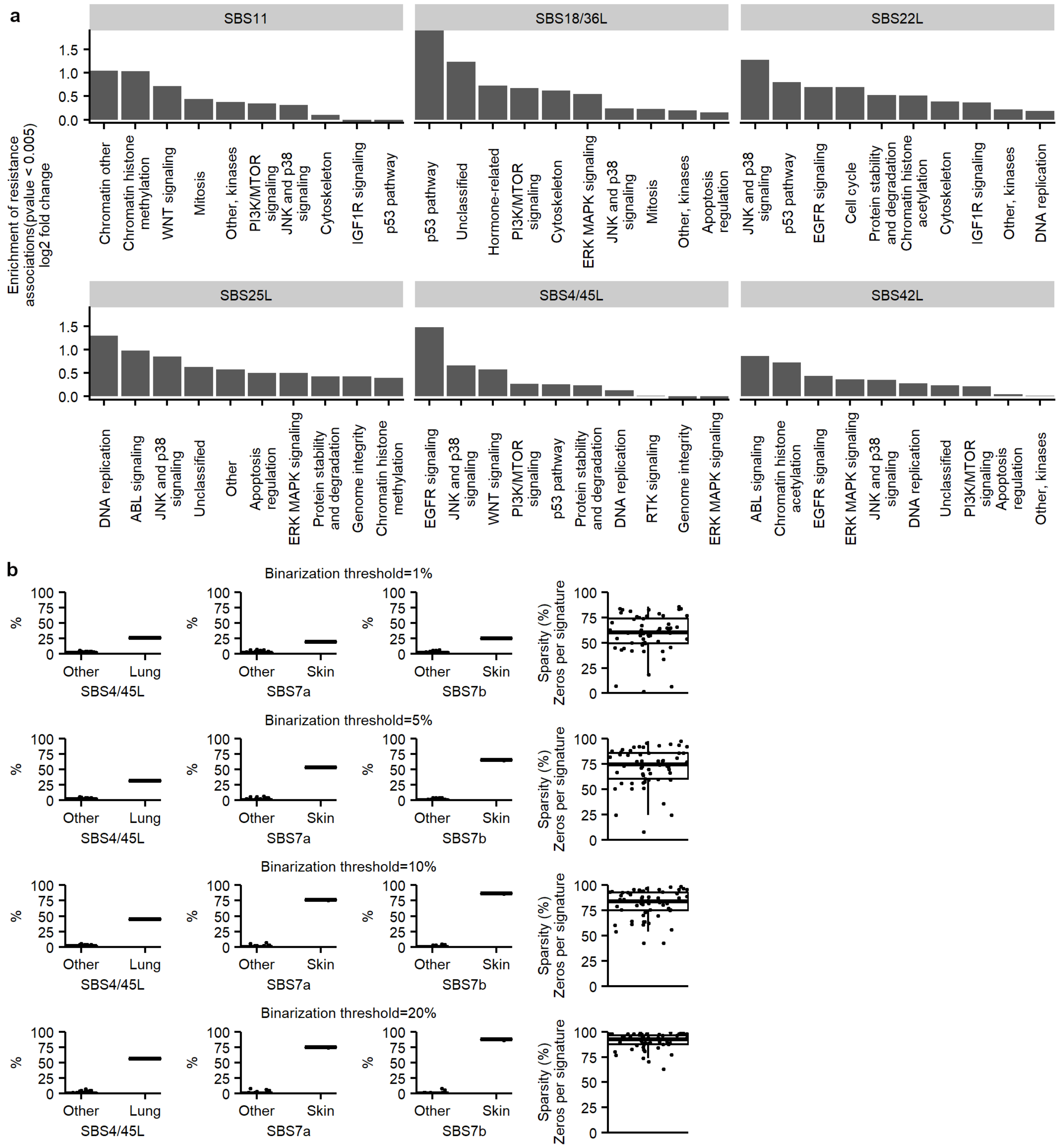

Supplementary Figure 15. Additional analyses concerning mutational signature markers. **a**, The log2 ratio of observed vs. expected frequencies of occurrence of drug target pathways in resistance associations identified via the replication test (p-value<0.005) for the top 6 signatures (by the number of resistance associations, see Fig. 6C) that were linked with various chemical

exposures. **b**, Different binarization thresholds ranging from 1% to 20% evaluated by the presence of signatures across 33 different tissues (after binarization) considering signatures that are known to be predominantly present only in certain tissues, i.e., the tobacco smoking signature SBS4/45L in lung and the UV signatures SBS7a and SBS7b in skin. For each threshold, we measured percent of cell lines per tissue that has the binarized exposure “1” for a signature and the sparsity of (all) signatures, expressed as percentage of zeros per signature. The center line of box plots denotes medians of all data points and the box hinges correspond to the 1st and 3rd quartiles, while whiskers extend to 1.5 × IQR from the hinges. Source data are provided as a Source Data file.

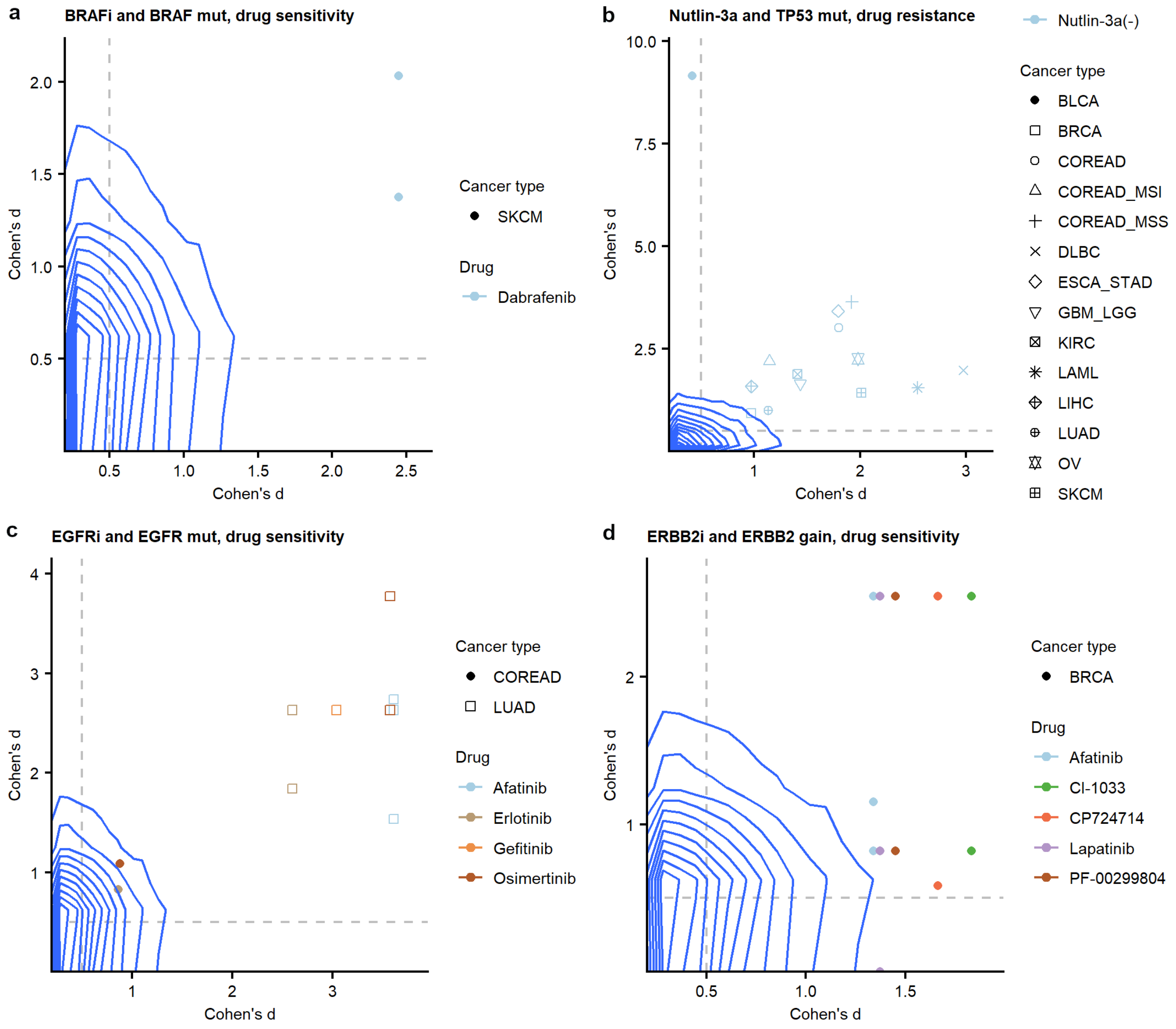

Supplementary Figure 16. Drug sensitivity associations from ”two-way” replication tests involving known positive controls. a, Sensitivity association between *BRAF* mutation and BRAF inhibitor dabrafenib in melanoma cells. **b**, Resistance associations between TP53 mutation and Nutlin-3a detected in several cancer types. **c**, Sensitivity associations between

EGFR mutation and EGFR inhibitors in colorectal and lung adenocarcinoma cells. **d**, Sensitivity association between *ERBB2* gain and several ERBB2 inhibitors in breast cancer cells. Blue lines are the contours of the 2D kernel density estimates of the distribution of associations between all drugs and mutations in all examined cancer genes (panels **a**, **b**, **c**) or all drugs and all recurrent copy number alterations (panel **d**). The dashed lines denote the 0.5 effect size threshold. Source data are provided as a Source Data file.

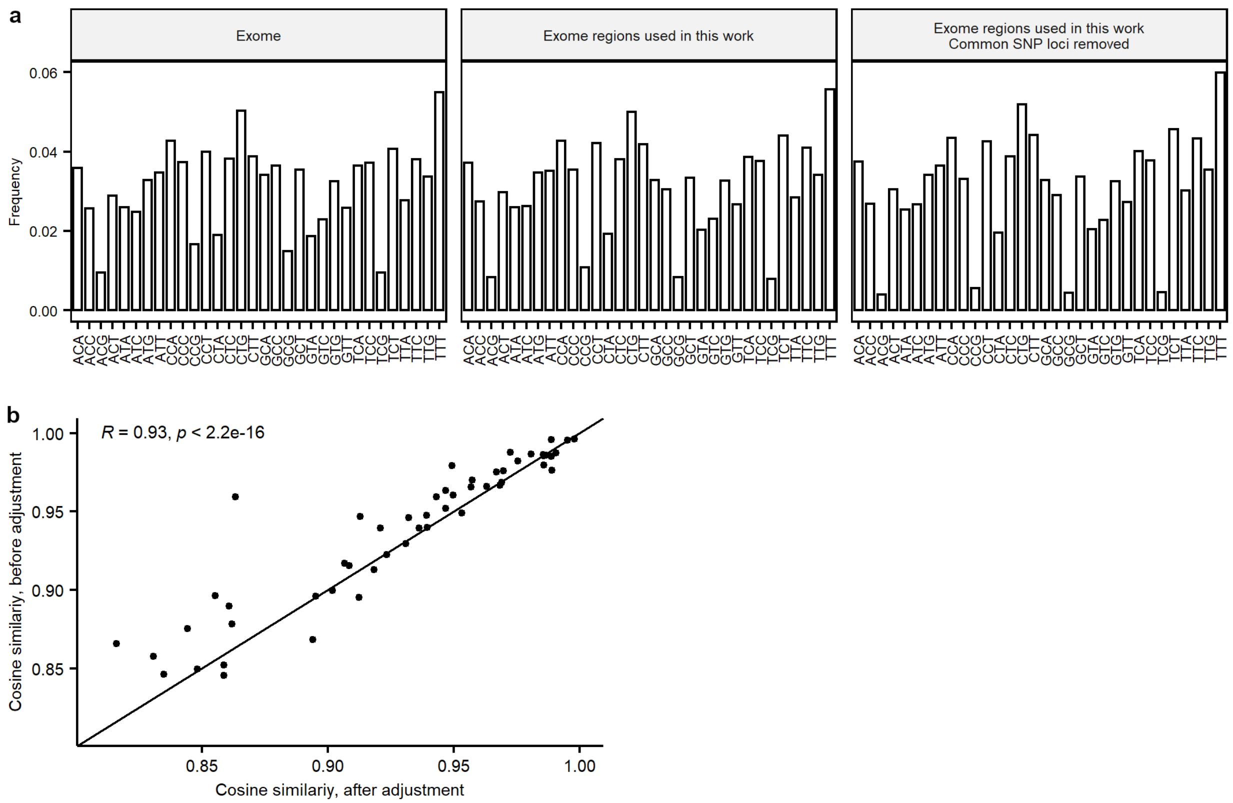

Supplementary Figure 17. Exome and genome composition effects on mutational signature spectra. a, The exome, exome regions and exome regions after removal of common SNP loci (i.e., filtering of the germline variants) have very similar trinucleotide composition, suggesting that the mutational signatures extraction was not biased in terms of the trinucleotide composition due to the filtering of the germline variants. **b**, Comparison of our mutational signatures extracted from the cell line exomes to the PCAWG signatures extracted from WGS tumor samples. The cell line signatures adjusted to match the WGS trinucleotide composition have similar cosine similarities to the PCAWG signatures as the unadjusted signatures (R denotes Pearson correlation; p denotes p-value of a two-sided t-test). The single point in the upper left quadrant whose cosine similarity is affected by adjustment to a larger extent than other points corresponds to the signature 49 (a possible artefact signature). Source data are provided as a Source Data file.
